# Manipulation of facet orientation in hybrid perovskite polycrystalline films by cation cascade

**DOI:** 10.1038/s41467-018-05076-w

**Published:** 2018-07-18

**Authors:** Guanhaojie Zheng, Cheng Zhu, Jingyuan Ma, Xiaonan Zhang, Gang Tang, Runguang Li, Yihua Chen, Liang Li, Jinsong Hu, Jiawang Hong, Qi Chen, Xingyu Gao, Huanping Zhou

**Affiliations:** 1 Beijing Key Laboratory for Theory and Technology of Advanced Battery Materials, Department of Materials Science and Engineering, College of Engineering, Peking University, 100871 Beijing, China; 2 Shanghai Synchrotron Radiation Facility, Shanghai Institute of Applied Physics, Chinese Academy of Sciences, 201204 Shanghai, China; 3 University of Chinese Academy of Sciences, 100049 Beijing, China; 4 Beijing Key Laboratory of Nanophotonics and Ultrafine Optoelectronic Systems, School of Materials Science and Engineering, Beijing Institute of Technology, 100081 Beijing, China; 5 Beijing National Laboratory for Molecular Science, Key Laboratory of Molecular Nanostructure and Nanotechnology, Institute of Chemistry, Chinese Academy of Sciences, 100190 Beijing, China; 6 School of Aerospace Engineering, Beijing Institute of Technology, 100081 Beijing, China; 7 State Key Laboratory for Advanced Metals and Materials, University of Science and Technology Beijing, No. 30 Xueyuan Rd, Haidian District, 100083 Beijing, China

## Abstract

Crystal orientations in multiple orders correlate to the properties of polycrystalline materials, and it is critical to manipulate these microstructural arrangements to enhance device performance. Herein, we report a controllable approach to manipulate the facet orientation within the ABX_3_ hybrid perovskites polycrystalline films by cation cascade doping at A-site. Two-dimensional synchrotron radiation grazing incidence wide-angle X-ray scattering is employed to probe the crystal orientations in multiple orders in mixed perovskites thin films, revealing a general pattern to guide crystal planes stacking upon extrinsic doping during crystallization. Different from previous studies, this method enables to adjust the crystal stacking mode of certain crystallographic planes in polycrystalline perovskites. Moreover, the preferred facet orientation is found to facilitate photocarrier transport across the absorber and pertaining interface in the resultant PV device, which provides an exemplary paradigm for further explorations that relate to the microstructures of hybrid perovskite materials and relevant optoelectronics.

## Abstract (web summary)

Crystal facet orientations of the polycrystalline hybrid lead halide perovskite thin films play a crucial role in determining the device performance. Here Zheng et al. demonstrate effective control of the crystal stacking mode by cation cascade doping, which promotes the charge transport in the photovoltaic device.

## Introduction

Metal halide perovskites with the general formula ABX_3_ are ideal candidates for a myriad of applications as a result of their unique optoelectronic properties, including large absorption coefficient, high mobility, and long diffusion length^1–6^. The state-of-the-art photovoltaic device based on perovskite materials features outstanding open-circuit voltage deficit and external quantum efficiency (EQE), approaching the commercially available c-Si counterpart^7,8^. To assure the best attainable power output, research efforts are allocated into two major aspects, one is to generate sufficient photocarriers by realizing high-quality perovskite crystals with prolonged carrier lifetime and/or optical confinement in the device configurations^9–12^, and the other is to improve external extraction efficiency of the photocarriers mostly at the adjacent contact by interface engineering^13–16^. These attempts lead to significant progresses in perovskite materials/devices in macroscopic scale toward high efficiency. Yet, it is less exploited in the micro/mesoscopic scale (e.g., intra-grain scale) for the hybrid perovskites, which is intuitively responsible for efficient photocarrier behavior in the materials and devices^17–19^.

A profound understanding and exquisite control of perovskite crystals in the context of microstructural arrangement is recently considered as an effective strategy to boost the photovoltaic efficiency of devices^18^. It was reported that the photoluminescence and carrier lifetimes varied between different grains within the same polycrystalline perovskite film, whereas the optoelectronic property of the inferior grains can be further activated to be superior with the assistance of appropriate chemical treatment^18^. The spatial heterogeneity within the perovskite polycrystalline film was later observed in terms of open-circuit photovoltage and short-circuit photocurrent mostly due to facet-dependent fluctuations in each individual grain^19^. It was claimed that facet-dependent variations of photovoltaic efficiency in individual grains of perovskite were ascribed to the anisotropic distributed trap densities that are orientation dependent. Though the microstructural arrangement of the polycrystalline is another decisive factor for device performance, it is not trivial to control the crystal orientation/facet in polycrystalline perovskite films, mainly because the hybrid nature of perovskites endorses extremely fast crystallization during film growth^20^. To date, most reported film growth methods readily produce perovskite polycrystalline films in different morphology^21–28^, but few of them provide anisotropic crystal orientation and pertinent facets in a controllable manner. Feasible approaches have been made to develop the perovskite film with preferred growth over a particular crystallographic plane, including precisely controlling the thermal gradient, modulating the intermediates, and exerting the external forces, etc^10,29–33^. One of these few examples include the fabrication of hybrid perovskite films with pure crystal orientation by using a thermal-gradient-assisted directional crystallization method^29^. An extraordinary carrier mobility was observed along with preferred horizontal direction in thick films (on the scale of few to tens μm). Another recent document reported the topotactic-oriented transformation for uniaxial-oriented perovskite films by introducing chlorine-contained precursors^10^. It observed a 300% higher carrier mobility in the resultant film than that in the reference, whereas the (−111) uniaxial orientation aligned perpendicular to the substrate. These indicate that the control on the chemical composition of the precursors or the preparation procedure could enable crystallization orientation or preferred growth over particular crystallographic planes, namely, the emergence of some dominant crystallographic plane(s) accompanying with the substantially decreased diffraction intensity of some other crystallographic plane(s). Yet, it lacks an effective approach to systematically adjust the crystal stacking (a substantial different crystal plane stacking along in-plane and out-of-plane directions) with respect to a certain crystallographic plane in polycrystalline perovskite thin films without morphological penalty, and further to correlate the thin film microstructure and photovoltaic properties.

In this contribution, we examine and manipulate the crystal facet orientation upon crystallographic plane stacking via cation cascade doping in the mixed perovskite thin films. Specifically, a spectrum of alkali elements (Cs, Rb, and K) is accumulatively introduced in sequence during film growth to create A-site cation cascade in FAMA-mixed perovskites. It results in a substantial crystal facet rotation along in-plane and out-of-plane directions by two-dimensional synchrotron radiation grazing incidence wide-angle X-ray scattering (GIWAXS) measurement. Moreover, the absorber with preferred facet orientation (we adopt Cs^+^-doping FAMA perovskite as an exemplary platform) exhibits higher carrier mobility along the film thickness and photocurrent in the corresponding device, which is ascribed to the efficient charge extraction accompanied with reduced carrier recombination possibly due to the facet contact. The optimized device based on mixed perovskites upon cation cascade achieves the power conversion efficiency (PCE) of 20.99% (stabilized efficiency of 20.41%). The cation cascade doping is proven to effectively adjust the crystal plane stacking, which is expected to further boost the photovoltaic device efficiency, and to find other potential applications such as photocatalysis, not restricted in thin films. These findings further illustrate the feasibility to tailor the multi-order of crystalline structure and consequent properties of hybrid perovskite materials in the intra-grain scale, which suggests an alternative direction to explore the relationship between microstructure/properties among wide-ranging optoelectronic semiconductors.

## Results

### Film characterization of X-ray diffraction (XRD) and scanning electronic microscopy (SEM)

The cation cascade doping in the mixed perovskites films has been created based on the (FAPbI_3_)_85_(MAPbBr_3_)_15_ (denoted as FAMA) perovskites, considering its phase stability at room temperature and decent photovoltaic efficiency in related devices^7,34^. The A-site substitutions are covered from FAMACs, FAMACsRb, to FAMACsRbK, by stepwise addition of A cations with continuously decreased ionic radii. Specifically, Cs^+^ was first doped in FAMA with the volume ratio of 1 to 12.5%, and 5% Cs-doped perovskites ((FAMA)_95_Cs_5_, denoted as FAMACs) were further served as the host for Rb and K doping, owing to its outstanding photovoltaic behavior among the full spectrum of Cs-doped perovskites, as evidenced in the later discussion. The corresponding films were obtained by the conventional one-step solution process with chlorobenzene as the antisolvent^34,35^, where the detailed procedure is shown in the experimental section.

**Fig. 1:**
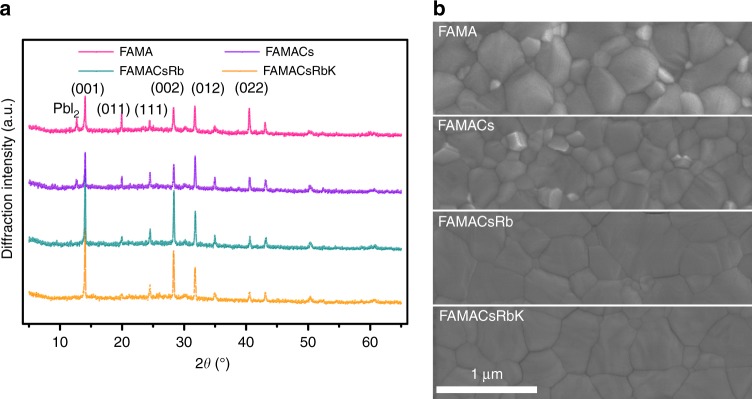
One-dimensional XRD and SEM image. **a** XRD pattern and **b** SEM images for perovskite films with cation cascade doping labeled as FAMA, FAMACs, FAMACsRb, and FAMACsRbK, respectively

The crystal phase and morphology of perovskite films with cation cascade has been extensively studied. Figure 1a shows the typical XRD patterns of perovskite films in the series of A cation cascade. It was found that all the mixed perovskite films display *α* black phase rather than *δ* non-perovskite phase, regardless of the doping ions and their concentrations. The major peaks at 14.04°, 19.94°, 24.52°, 28.3°, 31.74°, and 40.51° is assigned to (001), (011), (111), (002), (012), and (022) crystal planes, respectively^36^. The peak intensity between (001)/(002) is also consistent among all samples under investigation, indicating cation cascade does not result in significant preferable growth along certain crystalline planes given the above-mentioned crystal growth condition. However, the relative intensities of (011), (111), (012), and (022) varied randomly in these different samples, when compared with the corresponding (001) crystal planes, as indicated in Supplementary Table 1 and Supporting Note 1. This means that the crystal plane orientation in the perovskite film cannot be achieved from a simple cation cascade doping by changing the ion radii. SEM images of the cation cascade perovskite films are shown in Fig. 1b. All the films are uniform and highly crystalline with similar compact texture and the grain sizes are in the range of hundreds of nanometers. Upon the introduction of Cs^+^, the grain size slightly decreased which was in good agreement with previous works^37^. Interestingly, further addition of Rb^+^ or Rb^+^/K^+^ in FAMACs enhanced grain size in the as-prepared films. We would like to note that it is not the decisive parameter of the grain size in perovskite films to determine the pertinent device performance, since decent device efficiency has been reported for devices based on absorbers with either large or small grains^18,38–40^. We attempted to understand the film microstructure in intra-grain scale, as well as the relationship between multiple ordered crystal orientations and the optoelectronic properties.

### Microstructure analysis with GIWAXS measurement

**Fig. 2:**
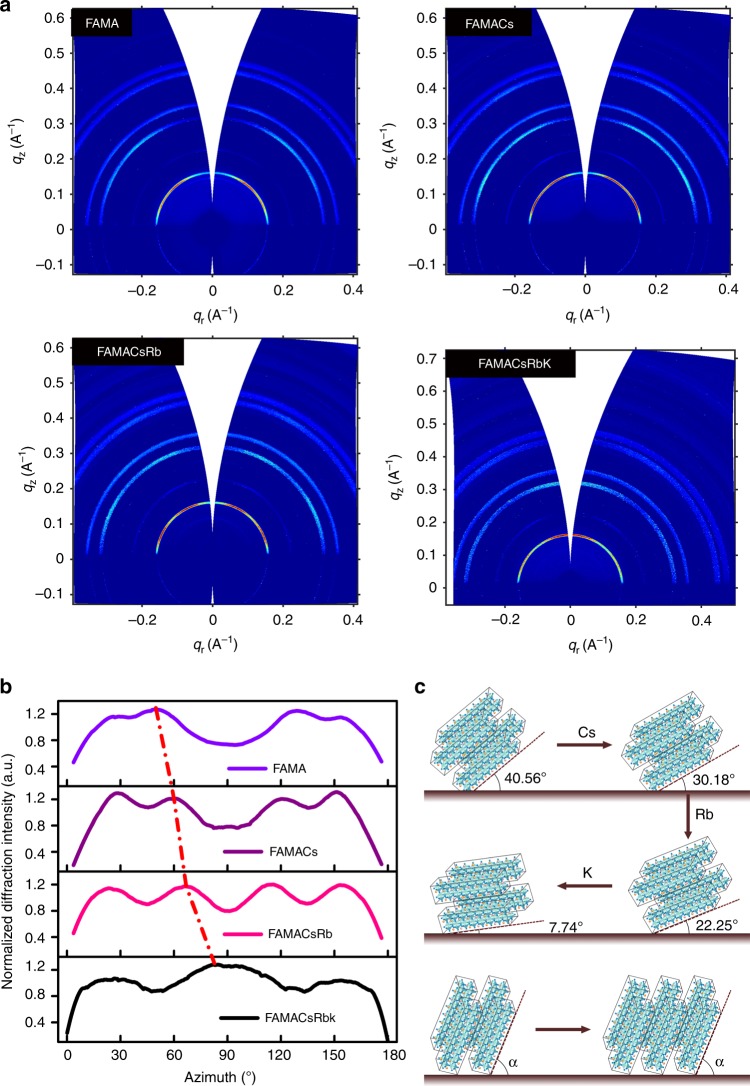
GIWAXS analysis and schematic diagram for microstructure evolution process. **a** GIWAXS patterns for perovskite films with cation cascade doping as FAMA, FAMACs, FAMACsRb, and FAMACsRbK, respectively. **b** Integrated intensity plots azimuthally along the ring at *q*_r_ approximate to 0.16 Å^−1^, assigned to the (001) plane of corresponding perovskite films noted in **a**. **c** The evolution schematic diagram of GIWAXS patterns occurred with cation cascade doping

Two-dimensional GIWAXS can precisely detect the crystal structure of the measured samples in microscale, e.g., the crystal orientations in multiple orders^30,31,41–43^. GIWAXS has been well implemented in the understanding of perovskite film orientation, including film formation process, the relationship between precursor and perovskite crystal orientation, lower dimensional perovskite, etc^31,43^. We conducted synchrotron radiation GIWAXS to probe the microstructure and the crystal orientation of the perovskite polycrystalline film in multiple orders with the cation cascade. Remarkably, it is found that the stacking pattern of crystal planes in the hybrid perovskites thin film changed along with A-site doping, particularly via cation cascade doping. Figure 2a depicts the GIWAXS patterns of the mixed perovskite films over cation cascade doping and the results derived. The corresponding diffraction mottling (red color section of the ring at *q*_r_ approximate to 0.16 Å^−1^) exhibits a strong diffraction signal for (001) plane with crystal plane stacking orientational preference, where **q** is the scattering vector in reciprocal space. It was observed clearly that the stacking patterns of preferred orientation along with (001) changed distinctly when the doping cations in the films extended from Cs, Rb, to K accumulatively. Moreover, we integrated the GIWAXS pattern azimuthally over the ring at *q*_r_ approximate to 0.16 Å^−1^. As depicted in Fig. 2b, the diffraction signal of (001) plane for the FAMA mainly displayed as a protuberance distributed from the azimuth angle of about 25°(180–25)° to 60°(180–60)° with indistinct peaks at 25°(180–25)° and 49.44°(180–49.44)°, which indicated a relatively disordered stacking due to the relatively wide distribution of *α* angle. When cesium was introduced, the protuberance split obviously and the indistinct diffraction mottling at the azimuth angle of 49.44°(180–49.44)° became more obvious and moved slightly toward the azimuth angle of 90°. It was found that except for the shift of diffraction mottling at the azimuth angle, the relative intensity at low azimuth became stronger and four peaks were found in the integration line. With the cation cascade doping based on FAMA perovskites, the diffraction mottling shifted from the original azimuth angle of 49.44° to 59.82° (FAMACs), 67.75° (FAMACsRb) then to 82.26° (FAMACsRbK) systematically. Meanwhile, the diffraction mottling at the azimuth angle of 25°(180–25)° shifted slightly to the lower azimuth angle accompanied with a stronger relative intensity. On the high azimuth angle side which is higher than 90°, the diffraction mottling follows an opposite trend, whereas the 130.56° peak shifted back toward 90° direction. In the context of crystal microstructure, the apparent evolution of the diffraction mottling indicates the changing preferential crystal orientation of the (001) planes due to the doping in the perovskites. Furthermore, high-order crystal planes (e.g., (011) and (002) planes) exhibit the change in staking pattern which is in self-consistency with that of (001) planes (Supplementary Fig. 1). The possible crystal stacking pattern of the presented hybrid perovskite materials are illustrated in Fig. 2c. Accordingly, it is visulized that the (001) crystal plane has the tendency from *α*-orientated stacking to both the in-plane and out-of-plane stacking with the cation cascade doping. It also indicates the enhanced long-range-orienated crystallization, which is beneficial for charge transport and consequently device performance, as will be dicussed later^10,29,44^. In addition, to investigate the crystal quality and orientation characteristics upon cation cascade doping, we integrated the intensity of Debye rings from GIWAXS data to obtain the diffraction intensity vs. 2-theta (Supplementary Fig. 2). The relative intensities of high orders with respect to that of (001) plane in doped samples differ from the theoretical value (Supplementary Table 2), implying possible changes in crystal orientation. Interestingly, the deviation of intensity ratio (high-order plane vs. (001) plane) in the perovskite films with cation cascade doping did not change much. Given the similar integral area of (001) peak in each sample, it indicates the series of perovskite films bear the comparable crystal quality and preferable growth planes.

To compare with intentional incorporation of both Rb^+^ and K^+^ into the FAMACs film in series, the FAMA films solely doped with either Rb^+^ or K^+^ were also investigated by GIWAXS, respectively. The (001) plane of the crystals visualized in the integrated plot from the GIWAXS patterns are shown in Supplementary Fig. 3. Interestingly, the resultant films showed limited diffraction mottling shift along azimuth angle with the individual addition of Rb^+^ or K^+^ into the FAMA films, which distinguished from that of samples doping with cation cascade. In addition, we excluded the effect of PbI_2_ with GIWAXS, which will be discussed in our following work in details. This substantiates the effectiveness of the cation cascade doping for the crystal orientation manipulation in polycrystalline perovskite films, whereas the underlying mechanism is not fully revealed yet. Most likely, the preference change observed in facet orientation could correlate to the crystal lattice distortion due to the series of dopants with different ionic radius^7,45^. The diffraction peak location of corresponding crystallographic planes in cascade-doping film is shown in Supplementary Table 3. And Supplementary Fig. 4 shows the XRD profiles (001 plane) of the polycrystalline film and the as-derived micro-strain within crystals. The extrinsic doping in mixed perovskites inevitably leads to the mismatch of crystal lattice constant, and thus crystal distortion to certain degree. It is reported the radii of A cation in perovskites can affect the B–X bonding length/angle, which obeys the Goldschmidt tolerance factor of ABX_3_ structure^46^. In the viewpoint of thermodynamics, Rb^+^ and K^+^ are probably excluded from the perovskite crystal structures if they are intended to be incorporated separately. However, the cation cascade leads to a gradual extension of the dopant size without abrupt change, which is likely to be kinetically favored to guide the preferred facet orientation during film crystallization. When the extrinsic cations were introduced as the perovskite precursor, the resulting films showed improved crystallinity. It indicates crystal planes stacking with higher regularity, as is observed from the obvious splitting of the swell presented in the integrated curves and the emergence of sharp peaks in the range of the azimuth angle between 0–180°. In addition, the larger the inoic radii difference between the doping cation and the host, the more significant changes it bears regarding the crystallagraphinc plane stacking. These observations indicate the cation cascade doping interferes with the crystallization kinetics to direct the microstructural arrangement of crystalline in perovskites thin films. According to previously suggested film growth model^47,48^, the bulk perovskite polycrystalline films were formed involving the intermediates of [PbI_6_]^4−^ polyiodide. The preferred orientation in the perovskite film is related to the corresponding nucleation dynamics, where specific rearrangement of the initial [PbI_6_]^4−^ octahedral framework occurred during the crystallization process, in order to reduce the overall chemical potential and form thermodynamically more stable perovskite structures. But it still requires indispensable efforts to fully understand the change of preferred orientation regarding the stacking of (001) crystal plane from *α* orientation toward the in-plane and out-of-plane induced by cation cascade doping. The proposed mechanism that governs the manipulation of crystal stacking pattern in the present work is, to some extent, similar with that for preferred crystal crystallographic orientations along particular direction in the perspective of interfered crystallization kinetics/thermodynamics mentioned above^10,29,32,33^. The cation cascade doping manipulates the crystal stacking orientation, which reveals the crystal orientations on a different level in terms of microstructural arrangements. Additionally, this method is rather simple and easy for operation, when compared to many other works based on precisely controlling the crystallization kinetics. The change in facet orientation provides an alternative approach to adjust the interface between perovskite thin films and the carrier transport layer, which ultimately determines the pertaining device performance as is discussed in the next section.

**Fig. 3:**
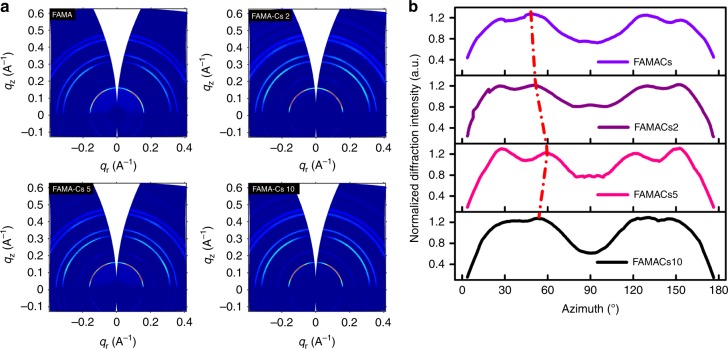
GIWAXS analysis for cesium-doped samples. **a** GIWAXS patterns for perovskite films with different cesium concentration labeled as FAMA-Cs 0, FAMA-Cs 2, FAMA-Cs 5, and FAMA-Cs 10. **b** Integrated intensity plots azimuthally along the ring at *q*_r_ approximate to 0.16 Å^−1^, assigned to the (001) plane of corresponding perovskite films corresponding to those in **a**

Subsequently, we adopted Cs^+^-doped perovskites as an exemplary platform to study the impact of crystal stacking orientation on the optoelectronic behavior of the resulting films and devices. We introduced different doping concentration of Cs^+^ in the perovskite precursor, from 0 to 12.5%. Due to the smaller ionic radius of Cs^+^ compared with MA^+^ or FA^+^, it achieved the preference of orientation in crystal plane stacking, whereas the distinct stacking orientations were observed along with (001) crystallographic planes (*q*_r_ approximate to 0.16 Å^−1^), as shown in Fig. 3a. The accumulation of facet orientation grows following the gradient of doping concentration. As shown in Fig. 3a, by increasing the cesium-doping concentration to 5%, the diffraction mottling moved toward high azimuth angle from 49.44° to 59.82°. Meanwhile, the relative intensity at low azimuth angle of Cs^+^-doped film became stronger and the integration line splitted into four peaks, as shown in Fig. 3b. When the concentration of Cs^+^ was increased to 10%, all peaks disappeared, and replaced with a convex protrusion, which indicated an evolution procedure from high long-range crystallinity to a relatively disordered state. Therefore, it clearly indicates that the crystal orientation of the presenting (001) plane are effectively manipulated by Cs^+^ doping in FAMA, reminiscent of the cation cascade doping discussed above. This also suggests that compared to other thermal gradient or the intermediate compound-assisted approaches that enables the preferred growth over a particular crystallographic plane, the cation cascade doping that is responsible for adjusting the crystal stacking pattern is proven to be adopted universally.

**Fig. 4:**
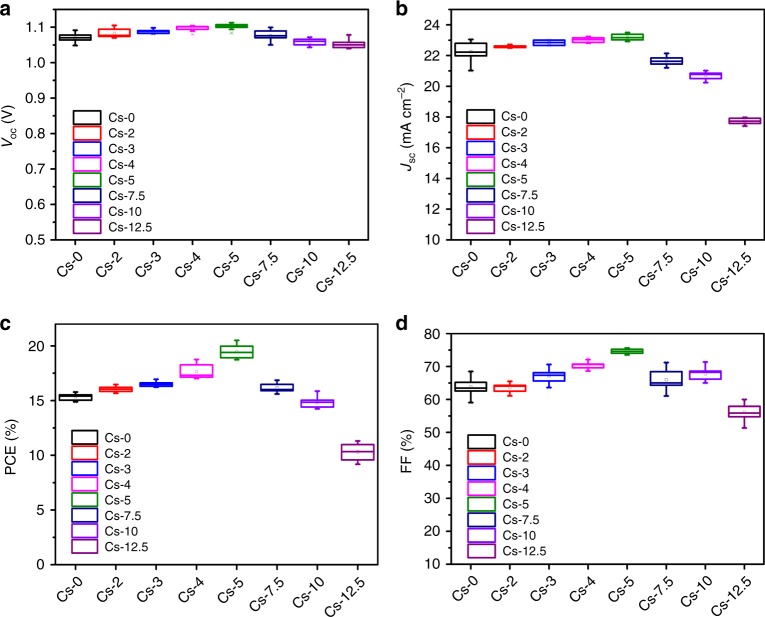
Statistics of photovoltaic parameters. Statistics of *I*–*V* performance parameters: *V*_oc_ (**a**), *J*_sc_ (**b**), PCE (**c**), FF (**d**) for devices based on mixed FAMA perovskites with different cesium-doping concentration. Twelve devices for each Cs^+^-doping concentration were involved in the statistics

To reveal how intra-grain crystal orientation interfere the carrier behavior within the photovoltaic cells, devices based on mixed perovskites with (FAMA)_(100−*x*)_Cs_*x*_ cascade were fabricated and investigated. We fixed the molar ratio of FAPbI_3_:MAPbBr_3_ at 85:15 according to previous studies^34^ with different Cs^+^-doping concentrations and implemented the as-prepared absorber in devices with the common configuration of ITO/SnO_2_/Perovskite/Spiro/Ag (Supplementary Fig. 5). The open-circuit voltage (*V*_oc_), short-circuit current (*J*_sc_), fill factor (FF), as well as PCE of the resulting devices are summarized in Fig. 4. It was found that the optoelectronic parameters, particularly *V*_oc_ and FF are maximized when the Cs^+^-doping concentration reached 5%. Accordingly, the average PCE increased from 15.35 to 19.49%. The typical devices performance parameters for perovskite films with 0, 5, and 10% cesium content were shown in Supplementary Table 4. Photocurrent density-voltage characteristics, stabilized efficiency, and EQE results are shown in Supplementary Fig. 6. These results clearly indicate that the incorporation of cesium enhances the performance of the mixed perovskite-based photovoltaic devices effectively.

The morphology, XRD pattern, UV-absorption, and photoluminescence of the Cs^+^-doped perovskite films were provided to evaluate the quality of the absorber itself. As displayed in Supplementary Fig. 7, the main phase and morphology of samples did not exhibit apparent differences, regardless of the Cs^+^ content. This is also in good agreement with the finding of above cation cascade doping. It should be mentioned the sample with low Cs^+^ amount showed a PbI_2_ peak at 12.8° in the corresponding XRD pattern (Supplementary Fig. 7b), while the presence of PbI_2_ residual in the perovskite film affects the photovoltaic performance of the resulting device in both ways, which has been intensively discussed elsewhere^49–51^. To avoid distracting the main focus of the present work, we have excluded the impact of PbI_2_ in the perovskite film on the device performance, as is discussed in the Supporting Information. Supplementary Fig. 7c confirmed that cesium incorporation did not enhance the film absorption systematically. However, there was a blue shift of the absorption edge with the increased cesium concentration, as shown in Supplementary Fig. 7d, which matched well with the slightly enlarged bandgap by Cs^+^ incorporation.^34^ These results imply that the absorbers are not affected by cation cascade doping in terms of several major attributes for photovoltaic efficiency in the device. Considering the significant change in crystal orientation induced by Cs^+^ doping, it is reasonable to further investigate the carrier behavior at the interface within the device to depict the correlation between device performance and the crystal microstructure in the polycrystalline absorber layer.

### Conductive atomic force microscopy (c-AFM) data analysis

**Fig. 5:**
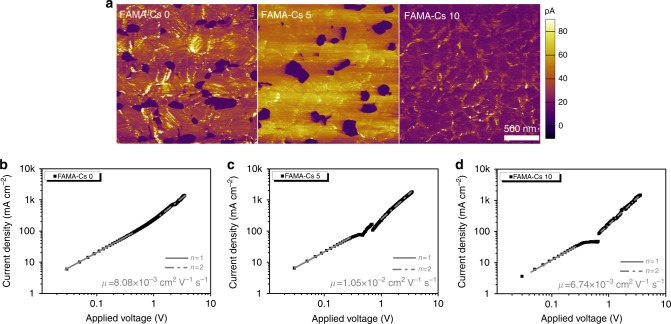
c-AFM and SCLC analysis for cesium-doped samples. **a** c-AFM images and SCLC data for FAMA perovskite films with different cesium-doping concentration (volume ratio: **b** 0, **c** 5 and, **d** 10%)

To correlate crystal orientation to photovoltaic efficiency in the solar cells, we carried out the Kelvin probe force microscopy (KPFM) (Supplementary Fig. 8 and Supplementary Table 5) and c-AFM measurements to study the local surface potential and film conduction through local current-voltage measurements, respectively. FAMACs film (5% cesium doping), representing the samples with preferred crystal orientation, was compared with the reference FAMA film. Figure 5a shows c-AFM images under a bias voltage of 2 V and the average photocurrent were listed in Supplementary Table 6. The dark region may be ascribed to PbI_2_, according to previous work^52^. In the reference, some lathy areas were observed with brighter contrast distributed sporadically, which was possibly caused by a specific orientation. We found that sample with optimized Cs^+^-doping concentration gave considerably larger currents in the entire measured area than that of the reference sample. When the concentration of Cs^+^ was increased to 10%, the c-AFM image showed an abrupt decreased conducting current which matched well with the enhanced disorder for 10% doped films, as shown in Fig. 3b, and the device performance (Fig. 4). These findings are also in coincidence with previous study that the conducting current flow in the perovskite film is facet dependent^19^. It suggests that the enhancement in conducting current flow is related to microstructure difference induced by cation cascade doping in the perovskites, with the preferred stacking of crystal planes.

### Space charge limited current (SCLC) measurements

The SCLC measurements are often employed to evaluate the carrier transport properties in perovskites materials^53^. The carrier mobility of the perovskites with preferred facet orientation has been extracted to link the optoelectronic properties to the microstructure of the polycrystalline film. Devices were fabricated with configurations of ITO/perovskites/Au to be operated in the trap-free SCLC regime above 0.4 V. And hole mobility was investigated in the single carrier device with the configuration of ITO/PEDOT:PSS/perovskites/P3HT/Au. The current density–voltage (*J*−*V*) characteristics were recorded under dark conditions, which was well fitted by the Mott–Gurney Law: *J* = (9/8)*ε*_r_*ε*_0_*μ*(*V*^2^/*L*^3^). The dielectric constant of 32 (measured in single crystal samples^29,41,54^) is adopted for fitting purpose, and *V* is the voltage drop across the device. The mobilities of the samples with different amount of Cs^+^ concentration (0, 5, and 10%) were calculated to be 8.08 × 10^−3^ cm^2^ V^−1^ s^−1^, 1.05 × 10^−2^ cm^2^ V^−1^ s^−1^, and 6.74 × 10^−3^ cm^2^ V^−1^ s^−1^, respectively (Fig. 5b). A higher mobility was observed with an enhancement of 30% in the perovskite sample with optimized cesium concentration compared with the reference, indicating substantial enhanced charge transportation in the thin films. The hole mobility in each sample follows the similar trend, as shown in Supplementary Fig. 9. This could be further correlated to the changes in microstructure of the perovskite film induced by Cs^+^ doping. It is reported that the carrier mobility along different crystal directions is varied, whereas the carriers travel along <112> and <200> crystallographic direction is faster than that of others^55^. In our study, the A-site doping did not change the preferred crystallographic planes, but their orientation. It clearly points out that the crystal stacking along different directions over the substrate could also interfere the carrier mobility of the perovskite films. The improved mobility in the FAMACs sample (5% Cs^+^ doped) is consistent with the enhanced current flow in the conductive AFM measurement as well. The optoelectronic properties were impacted upon A-site cation doping partially governed via manipulation of crystal facet orientation, which thus suggests an alternative way to boost the device performance.

### TPV, TPC, EIS, and IMPS measurements

**Fig. 6:**
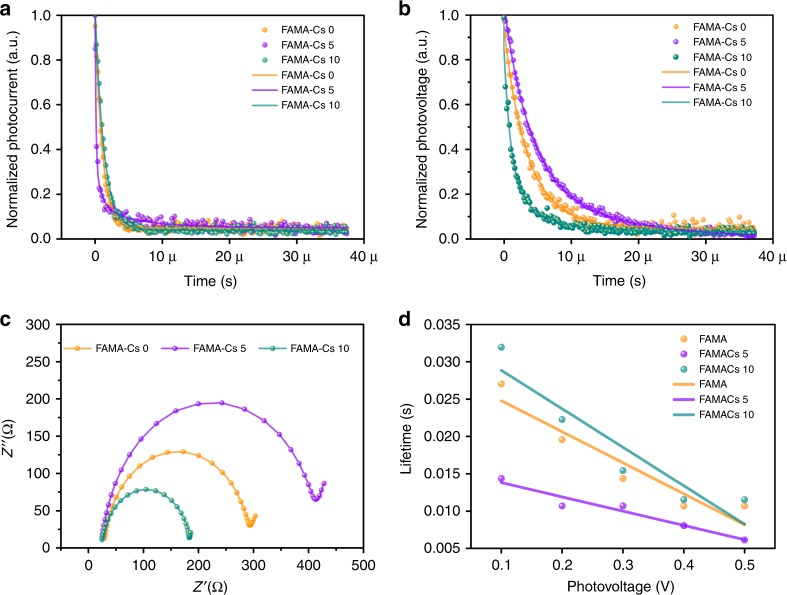
Analysis of carrier dynamics. **a** TPC, **b** TPV, **c** EIS, and **d** IMPS analysis for FAMA perovskite samples with different cesium-doping concentration (volume ratio: 0, 5, and 10%)

To further examine the effects of crystal facet orientation on the carrier dynamics within the device, we have conducted transient photovoltage (TPV) and transient photocurrent (TPC) measurements on the cells. In TPV measurements, devices are excited by a short laser pulse to receive a TPV response, which are widely employed to probe the electron lifetime related to recombination rates in the perovskites solar cells^13,56,57^. We compared three devices based on the absorbers of (FAMA)_(100−*x*)_Cs_*x*_ mixed perovskites with different orientation preference, whereas Cs contents are 0, 5, and 10%, respectively. TPC results on the devices were shown in Fig. 6a (fitted data in Supplementary Table 7). Accordingly, the (FAMA)_95_Cs_5_-based device obtained the shortest decay time of 0.35 μs, which indicated an enhanced charge transport process compared with that of a decay time of 1.33 μs, and 2.81 μs in reference sample and (FAMA)_90_Cs_10_-based device, respectively. Based on the voltage decay time for each device, the device with the 5% Cs^+^ doping exhibited the longest electron lifetime of 5.36 μs (Fig. 6b, fitted data in Supplementary Table 7). The increase of photovoltage decay time in the (FAMA)_95_Cs_5_-based device is probably attributed to the preferred orientation that enables improved carrier flow between perovskites and carrier transport layers. In addition, electrochemical impedance spectroscopy (EIS) characterization was performed to demonstrate carrier transport processes under illumination at the interface, as shown in Fig. 6c. We used various simple lumped RC circuit to satisfactorily model impedance responses of the cells. In the present planar perovskite cells, the middle frequency zone of EIS semicircle should be dominated by junction capacitance and recombination resistance related to the interfaces between transport materials and perovskite. According to Fig. 6c, the (FAMA)_95_Cs_5_-based device has the largest impedance among the three samples. The larger impedance indicates a substantial suppressed recombination in the (FAMA)_95_Cs_5_-based device, which probably originated from the improved carrier flow between perovskites and carrier transport layers. Furthermore, we carried out the intensity-modulated photocurrent spectroscopy (IMPS) measurement to reveal the kinetics of carrier extraction within the devices (Fig. 6d). As can be seen, devices derived from the optimized cesium concentration (5%) showed the shortest lifetime, indicating the most efficient carrier extraction and transfer process occurred. This is consistent with our TPV/TPC measurements, which thus reinforces our claim that the orientation variation induced by cesium doping substantially impact the carrier transport or extraction dynamics within the perovskite films and devices. Combined TPV, TPC, EIS, IMPS data with results mentioned in GIWAXS, KPFM, and J–V measurement sections, we argue that appropriate incorporation of Cs^+^ boosts device performance by taking effects of favored crystal orientation for improved interface and interfered crystal growth for better crystal quality.

## Discussion

The full characterization and devices optimization are well conducted on the Cs-doped FAMA compound, while extending toward Rb and K devices would be very helpful to understand the mixed perovskites beyond Cs. Accordingly, we carried out the full characterizations on the cascade-doping system, including energy-dispersive X-ray spectroscopy, X-ray photoelectron spectroscopy (XPS), c-AFM, SCLC, TPV, TPC, and IMPS. EDS and XPS (Supplementary Fig. 10 and Supplementary Table 8) displayed the spatial distribution of Cs, Rb, K in the samples. c-AFM measurement was used to study the film conductivity. Similar to the FAMA host doping with Cs^+^, we found that the currents increased following the cascade series, namely, FAMA<FAMACs<FAMACsRb<FAMACsRbK in the entire measured area, as shown in Supplementary Fig. 11 and Supplementary Table 9. This is in good agreement to the enhanced current in perovskite films upon Cs doping at optimized concentration. It thus confirms that the enhancement in photocurrent flow accompanying with cation cascade doping in the perovskites is extended beyond Cs doping, which is possibly associated to microstructure difference with the preferred stacking of crystal planes. Furthermore, SCLC measurements were employed to evaluate the carrier transport properties in perovskites materials with the configuration of ITO/perovskites/Au. The mobilities of the samples with cation cascade doping were calculated to be 0.37 × 10^−1^, 1.9 × 10^−1^, 7.7 × 10^−1^, and 8.5 × 10^−1^ cm^2^ V^−1^ s^−1^ for FAMA, FAMACs, FAMACsRb, and FAMACsRbK, respectively (Supplementary Fig. 12). It is clear that cation cascade doping results in a continuous increase in the mobility of the perovskite thin films, indicating substantial enhanced charge transportation in the absorber. It is consistent to the c-AFM measurement results, wherein the photocurrent increases according to the same pattern. The observation here further supports our previous argument holds in cesium-doping section, wherein the cascade doping in perovskite is able to control microstructure via manipulation of crystal facet orientation to influence the resultant film property. Besides, TPV and TPC measurements were employed to probe the photocarrier lifetime related to carrier kinetics in perovskites solar cells (Supplementary Fig. 13; fitted data in Supplementary Table 10). The FAMACsRbK-based device exhibited the shortest decay time of 0.45 μs, standing for the fastest charge transport process as compared with that of FAMACsRb, FAMACs, and FAMA-based devices (0.66, 0.89, and 1.21 μs, respectively). The decrease in photocurrent decay time indicated that the cascade doping effectively promoted the charge extraction and transport process which correlated perfectly with the orientation variation. IMPS result was shown in Supplementary Fig. 13d, where the *τ* was monotonically decreased from FAMACsRbK, to FAMACsRb, FAMACs, and FAMA, indicating a gradually enhanced charge extraction and transfer processes within perovskite films with cascade doping. Thus, we can confirm that the charge dynamics within the devices have been influenced by the crystal orientation even under different illumination intensity.

However, the change of carrier recombination dynamics in the FAMA, FAMACs, FAMACsRb, FAMACsRbK samples do not follow the same trend as compared to that of carrier transport. As shown in Supplementary Fig. 13b, the photovoltage decay time gradually increased first, namely, about 3.61 μs (FAMA), 5.36 μs (FAMACs), and 6.84 μs (FAMACsRb), but it unexpectedly decreased to about 4.0 μs (FAMACsRbK). As photovoltage decay time describes carrier recombination kinetics, it indicates that the underlying mechanism to govern carrier recombination may be complex. Cation cascade may introduce not only crystal orientation change but other effects, and they both affect the carrier recombination process at the interface. In addition, as shown in Supplementary Fig. 13c (EIS results), the FAMACsRb-based device has the largest impedance among the three samples, followed by FAMACs, FAMACsRbK, and FAMA. This is in excellent agreement with the above TPV results, where FAMACsRb instead of FAMACsRbK-based sample presents a substantial suppressed recombination.

The PCE, *V*_oc_, *J*_sc_, and FF of the resulting devices are summarized in Supplementary Figs. 14, 15 and Supplementary Table 11. It was clearly observed that the average PCE were enhanced distinctly when the doping cations in the films extended from FAMA to Cs and Rb accumulatively. It is in accordance with the recent report^7^ and our previous arguments. Interestingly, samples based on K-doping perovskite showed a slightly lower average PCE than that of Rb. The optimized device based on mixed perovskites upon cation cascade achieves the scanned PCE of 20.99% (stabilized efficiency of 20.41%) (Supplementary Fig. 16), which is among the highest efficiency of perovskite solar cell based on planar heterojunction configuration^58,59^. We attributed the maximized average device efficiency in the FAMACsRb to the simultaneously improved carrier transport and suppressed carrier recombination. The slightly dropped PCE in the FAMACsRbK when compared to FAMACsRb is probably rooted in the trade-off between the improved carrier transport and enhanced carrier recombination. Therefore, it is inferred the cascade doping upon K involvement is likely to introduce some secondary effect that affects the carrier behavior across the entire device, probably associated to the occupancy of K that may not necessarily stay in the crystal lattice. However, this topic is beyond our research scope of this work. Based on the full characterization on two series of samples, we have provided convincing results showing that the crystal orientation variation in the form of crystal plane stacking can definitely affect the carrier transport/extraction in the film/device. Further simulation results reveal that the anisotropic carrier transport in perovskite crystals, namely, different electron/hole effective masses along different crystallographic direction. Detailed calculation regarding the relationship between the crystal orientation variation and the microscopic transport has been provided in the Supplementary Table 12. We have thus demonstrated that the GIWAXS helps to reveal the microstructures of polycrystalline thin films, which correlates to the carrier transport that affects the device performance.

In summary, we carefully investigated the preferred facet orientation in mixed perovskites absorber by cation cascade doping, and adopted Cs^+^-doped perovskites as an exemplary platform to study the impacts of crystal stacking orientation on the optoelectronic behavior of the resulting absorber and devices. The GIWAXS measurements clearly revealed that the alkali elements doping effects on the crystal facet rotation with preferred orientation can be conducted in a controllable manner by doping concentration. The absorber layer with preferred facet orientation exhibits higher mobility across the film thickness as measured by c-AFM and SCLC measurements, indicating the preferred facet orientation accompanied with long-range crystalline order is able to influence the optoelectronic properties. Furthermore, the facet orientation facilitates the charge transport over the relevant interfaces, as was elucidated by TPV, TPC, EIS, and IMPS measurements. These add up to contribute to the improved photovoltaic efficiency in the resultant devices. Particularly, although the preferred crystallographic planes did not change upon cation cascade doping, their stacking mode with respect to a certain crystallographic plane still substantially impact the optoelectronic property of the film and devices. Our results provide a complete explanation in the perspective of device physics to the mechanism that governs the device performance in the mixed perovskites with A-site doping. The cation cascade doping thus serves as a feasible and effective tool for microstructural control in the hybrid perovskites to further break through the bottleneck on perovskite solar cell efficiencies.

## Methods

### Materials

All the commercial materials were used as received, including chlorobenzene (99.9%, Aladdin Industrial Corporation), hydrogen iodide (57%, Alfa Aesar), ethanol (AR Beijing Chemical Works), PbI_2_ (99.999%, Sigma-Aldrich), *N*,*N*-dimethylformamide (99.99%, Sigma-Aldrich), dimethyl sulfoxide (99.50%, Sigma-Aldrich), PbBr_2_ (99.9%, Aladdin Industrial Corporation), CsI (99.90%, Aladdin Industrial Corporation), RbI (99.90%, Aladdin Industrial Corporation), KI (99.90%, Aladdin Industrial Corporation), spiro-OMeTAD (Xi’an Polymer Light Technology Corp.), 4-tertbutylpyridine (99.90%, Sigma-Aldrich), lithium bis(trifluoromethylsulphonyl)imide (99.95%, Sigma-Aldrich), and ITO substrates. The HC(NH_2_)_2_I and CH_3_NH_3_Br were prepared according to procedure mentioned in previous work.

### Preparation of SnO_2_ nanoparticle film

The SnO_2_ colloid precursor was purchased from Alfa Aesar (tin (IV) oxide, 15% in H_2_O colloidal dispersion). The precursor were diluted by H_2_O to 2.67% before used. The final solution was spin coated onto glass/ITO substrates at 3000 rpm for 30 s, and then baked on a hot plate at 150 °C for 30 min in air.

### Perovskite precursor solutions

The precursor solutions were prepared according to the work delivered by Saliba^34^.

FAMA perovskite solution: The mixed perovskite solution were prepared by mixing FAI (1 M), PbI_2_ (1.1 M), MABr (0.2 M), PbBr_2_ (0.22 M) in anhydrous DMF:DMSO 4:1 (v:v), according to previous work with a slight amount of excessive PbI_2_. To be convenient, we labeled the mixed perovskite solution with compositions mentioned above as FAMA.

CsI, RbI, and KI solutions: CsI solution was deposited by dissolving CsI in pure DMSO with the concentration of 1.5 M; similarly, RbI and KI were predissolved as a 1.0 M stock solution in DMSO.

(FAMA)_(100__−__*x)*_Cs_*x*_ perovskite solution: (FAMA)_(100−*x*)_Cs_*x*_ perovskite solution was obtained by adding appropriate amount of CsI into 300 μL FAMA perovskite solution with different cesium concentrations (volume ratio, *x* = 100% × *V*_CsI_/(*V*_CsI_ + 300)) to achieve the desired cation composition from 0 to 12.5%. And we labeled the composition with optimized cesium concentration as FAMACs for short.

(FAMACs)_100__−__*x*_Rb_*x*_ and (FAMACsRb)_100__−__*x*_K_*x*_ perovskite solutions: (FAMACs)_100−*x*_Rb_*x*_ solution was obtained based on the (FAMA)_95_Cs_5_ perovskite solution by adding different concentration of RbI (volume ratio). Composition with optimized rubidium concentration was labeled as FAMACsRb. Similarly, (FAMACsRb)_100−*x*_K_*x*_ solution was prepared by adding KI (1 M) solution into FAMACsRb and the finally optimized composition was labeled as FAMACsRbK.

### Sample preparation and devices fabrication

The ITO substrate was sequentially washed with distilled water, acetone, ethanol, and isopropanol. Then, diluted SnO_2_ precursor was spin coated onto glass/ITO substrate, and annealed at 150 °C for 30 min in air atmosphere. For the mixed A-cation ABX_3_ metal halide perovskite layer, the one-step method that used chlorobenzene as an antisolvent developed by Saliba^34^ was adopted. In detail, the perovskite solutions with different composition were spin-coated in a two-step program at 1000 and 5000 rpm for 10 and 30 s, respectively. During the second step, 200 μL of chlorobenzene was poured on the spinning substrate 15 s prior to the end of the program. Then, the as-fabricated film were baked at 100 °C for 1 h in a nitrogen filled glove box. After the perovskite annealing, 30 μL Spiro-OMeTAD solution doped with LiTFSI and tBP was deposited at 2000 rpm for 45 s. The hole transport material solution was prepared by dissolving 80 mg spiro-OMeTAD, 30 μL 4-tert-butylpyridine, and 35 μL Li-TFSI/acetonitrile (260 mg mL^−1^) in 1 mL chlorobenzene. Finally, 100 nm Ag was thermally evaporated as counter electrode under a pressure of 5 × 10^−5^ Pa on top of the hole transport layer to form the back contact.

### Characterization

*J*–*V* characteristics of photovoltaic devices were measured in air at room temperature by using a Keithley 2400 source meter under simulated sunlight from an Oriel 300 solar simulator. The light intensity was calibrated by means of a KG-5 reference silicon solar cell. For the *J*–*V* measurement, the effective area of each cells was 0.102 cm^2^ calibrated by mask for all the solar cells involved in this work. The mask is a meal sheet with rectangular aperture of 0.173 × 0.59 cm^2^. The *J*–*V* results for the FAMA, FAMACs, FAMACsRb, FAMACsRbK-based devices were obtained with slow scan rate of 40 mV s^−1^ with 0.02 V step and 0.5 s delay time, which is usually adopted by the third party for certification. A cold field-emission SEM (Hitachi S-4800) was used to characterize top view morphology of different perovskite thin films. One-dimensional XRD spectra was obtained by using Rigaku D/MAX 2400 diffractometer with Cu K˛ radiation. Two-dimensional synchrotron radiation GIWAXS was performed at BL14B beamline, Shanghai synchrotron Radiation Facility with a wavelength of 0.6887 Å to analyze the crystallinity and orientation of the perovskite films^41^. Two-dimensional GIWAXS data were acquired by using a MarCCD with a distance c.a. 300 mm from the samples. The grazing incidence angle was fixed at 0.2° and the exposure time was set to 40 s. EQEs were carried out by an EnLi Technology (Taiwan) EQE measurement system calibrated against a certified reference silicon solar cell. EIS was executed under illumination by Zahner electrochemical Workstation. For the frequency response techniques, all the devices were measured under exactly the same condition, using frequency parameter from 1 MHz to 100 Hz. The illumination of 0.3 Sun was provided by a white light and 5 mV was set as the amplitude of sinusoidal modulation light intensity. TPC and TPV spectra were obtained by using a pulsed double frequency Nd:YAG laser (Brio, 20 Hz), at 532 nm with an ultra-low light intensity and a sub-nanosecond resolved digital oscilloscope (Tektronix DPO 7104) with input impedances of 1 MW and 50 W, respectively. The pulse duration is about 4 ns. KPFM was conducted using a MFP-3D bio (Oxford Instruments Asylum Research Inc.) with a NSG01/Pt probe. c-AFM was measured with a bias voltage of 2 V under illumination using the same instrument as KPFM. All the measurements of the solar cells were carried out in ambient air at room temperature without encapsulation.

### Data availability

The data that support the findings of this study are available from the corresponding author upon request.

## Electronic supplementary material

Supplementary Information

## References

[1] Rong Y (2015). Beyond efficiency: the challenge of stability in mesoscopic perovskite solar cells. Adv. Energy Mater..

[2] You J (2016). Improved air stability of perovskite solar cells *via* solution-processed metal oxide transport layers. Nat. Nanotech..

[3] Gratzel M (2014). The light and shade of perovskite solar cells. Nat. Mater..

[4] Green MA (2014). The emergence of perovskite solar cells. Nat. Photon..

[5] Meng L (2015). Recent advances in the inverted planar structure of perovskite solar cells. Acc. Chem. Res..

[6] Ponseca CS (2014). Organometal halide perovskite solar cell materials rationalized: ultrafast charge generation, high and microsecond-long balanced mobilities, and slow recombination. J. Am. Chem. Soc..

[7] Michael Saliba TM (2016). Incorporation of rubidium cations into perovskite solar cells improves photovoltaic performance. Science.

[8] Zhang W, Eperon GE, Snaith HJ (2016). Metal halide perovskites for energy applications. Nat. Energy.

[9] Liu Z (2017). Chemical reduction of intrinsic defects in thicker heterojunction planar perovskite solar cells. Adv. Mater..

[10] Kim DH (2017). 300% enhancement of carrier mobility in uniaxial-oriented perovskite films formed by topotactic-oriented attachment. Adv. Mater..

[11] Zheng G (2017). The investigation of an amidine-based additive in the perovskite films and solar cells. J. Semicond..

[12] Pellet N (2014). Mixed-organic-cation perovskite photovoltaics for enhanced solar-light harvesting. Angew. Chem. Int. Ed. Engl..

[13] Zhou H (2014). Interface engineering of highly efficient perovskite solar cells. Science.

[14] Dymshits A (2015). The electronic structure of metal oxide/organo metal halide perovskite junctions in perovskite based solar cells. Sci. Rep..

[15] Kim JH (2015). High‐performance and environmentally stable planar heterojunction perovskite solar cells based on a solution-processed copper-doped nickel oxide hole-transporting layer. Adv. Mater..

[16] Cho KT (2017). Highly efficient perovskite solar cells with a compositionally engineered perovskite/hole transporting material interface. Energy Environ. Sci..

[17] Huang F (2017). Effect of the microstructure of the functional layers on the efficiency of perovskite solar cells. Adv. Mater..

[18] deQuilettes DW (2015). Solar cells. Impact of microstructure on local carrier lifetime in perovskite solar cells. Science.

[19] Leblebici SY (2016). Facet-dependent photovoltaic efficiency variations in single grains of hybrid halide perovskite. Nat. Energy.

[20] Liang PW (2014). Additive enhanced crystallization of solution-processed perovskite for highly efficient planar-heterojunction solar cells. Adv. Mater..

[21] Zhou Y (2016). Exceptional morphology-preserving evolution of formamidinium lead triiodide perovskite thin films via organic-cation displacement. J. Am. Chem. Soc..

[22] Sharenko A (2015). Relationships between lead halide perovskite thin-film fabrication, morphology, and performance in solar cells. J. Am. Chem. Soc..

[23] Li L (2016). The additive coordination effect on hybrids perovskite crystallization and high-performance solar cell. Adv. Mater..

[24] Perez-Del-Rey D (2016). Strontium insertion in methylammonium lead iodide: long charge carrier lifetime and high fill-factor solar cells. Adv. Mater..

[25] Momblona C (2016). Efficient vacuum deposited p-i-n and n-i-p perovskite solar cells employing doped charge transport layers. Energy Environ. Sci..

[26] Xiao Z (2014). Efficient, high yield perovskite photovoltaic devices grown by interdiffusion of solution-processed precursor stacking layers. Energy Environ. Sci..

[27] Pang S (2016). Transformative evolution of organolead triiodide perovskite thin films from strong room-temperature solid-gas interaction between HPbI_3_-CH_3_NH_2_ precursor pair. J. Am. Chem. Soc..

[28] Zhou Y (2015). Microstructures of organometal trihalide perovskites for solar cells: their evolution from solutions and characterization. J. Phys. Chem. Lett..

[29] Cho N (2016). Pure crystal orientation and anisotropic charge transport in large-area hybrid perovskite films. Nat. Commun..

[30] Giesbrecht N (2016). Synthesis of perfectly oriented and micrometer-sized MAPbBr_3_ perovskite crystals for thin-film photovoltaic applications. ACS Energy Lett..

[31] Hu Y (2016). Hybrid perovskite/perovskite heterojunction solar cells. ACS Nano.

[32] Li, W. et al. Aquointermediate assisted highly orientated perovskite thin films toward thermally stable and efficient solar cells. *Adv. Energy Mater*. **121**, 28443–28453 (2017).

[33] Kim, W. et al. Oriented grains with preferred low-angle grain boundaries in halide perovskite films by pressure-induced crystallization. *Adv. Energy Mater*. **8**, 1702369 (2017).

[34] Saliba M (2016). Cesium-containing triple cation perovskite solar cells: improved stability, reproducibility and high efficiency. Energy Environ. Sci..

[35] Jeon NJ (2014). Solvent engineering for high-performance inorganic–organic hybrid perovskite solar cells. Nat. Mater..

[36] Zhumekenov AA (2016). Formamidinium lead halide perovskite crystals with unprecedented long carrier dynamics and diffusion length. ACS Energy Lett..

[37] Kulbak M (2015). Cesium enhances long-term stability of lead bromide perovskite-based solar cells. J. Phys. Chem. Lett..

[38] Yun JS (2015). Benefit of grain boundaries in organic-inorganic halide planar perovskite solar cells. J. Phys. Chem. Lett..

[39] Nie W (2015). High-efficiency solution-processed perovskite solar cells with millimeter-scale grains. Science.

[40] Ahn N (2015). Highly reproducible perovskite solar cells with average efficiency of 18.3% and best efficiency of 19.7% fabricated *via* Lewis base adduct of lead (II) iodide. J. Am. Chem. Soc..

[41] Wang ZK (2016). High efficiency Pb-In binary metal perovskite solar cells. Adv. Mater..

[42] Huang W (2015). Probing molecular and crystalline orientation in solution-processed perovskite solar cells. Adv. Funct. Mater..

[43] Schlipf, J., Müller-Buschbaum, P. Structure of organometal halide perovskite films as determined with grazing-incidence X-ray scattering methods. *Adv. Energy Mater*. **5**, 1700131 (2017).

[44] Liu S (2016). Imaging the long transport lengths of photo-generated carriers in oriented perovskite films. Nano Lett..

[45] Rehman W (2017). Photovoltaic mixed-cation lead mixed-halide perovskites: links between crystallinity, photo-stability and electronic properties. Energy Environ. Sci..

[46] Deepa M (2017). Cesium power: low Cs^+^ levels impart stability to perovskite solar cells. Phys. Chem. Chem. Phys..

[47] Hu Q (2017). In situ dynamic observations of perovskite crystallisation and microstructure evolution intermediated from [PbI_6_]^4−^ cage nanoparticles. Nat. Commun..

[48] Boopathi KM (2016). Synergistic improvements in stability and performance of lead iodide perovskite solar cells incorporating salt additives. J. Mater. Chem. A.

[49] Kim YC (2016). Beneficial effects of PbI_2_ Incorporated in organo-lead halide perovskite solar cells. Adv. Energy Mater..

[50] Chen Q (2014). Controllable self-induced passivation of hybrid lead iodide perovskites toward high performance solar cells. Nano Lett..

[51] Jacobsson TJ (2016). Unreacted PbI_2_ as a double-edged sword for enhancing the performance of perovskite solar cells. J. Am. Chem. Soc..

[52] Li JJ (2015). Microscopic investigation of grain boundaries in organolead halide perovskite solar cells. ACS Appl. Mater. Interfaces.

[53] Zhou N (2017). CsI pre-intercalation in the inorganic framework for efficient and stable FA_1-x_Cs_x_PbI_3_(Cl) perovskite solar cells. Small.

[54] Huang Y (2017). The intrinsic properties of FA_*(1−x)*_MA_*x*_PbI_3_ perovskite single crystals. J. Mater. Chem. A.

[55] Niu G (2016). Controlled orientation of perovskite films through mixed cations toward high performance perovskite solar cells. Nano Energy.

[56] Jiang Q (2016). Enhanced electron extraction using SnO_2_ for high-efficiency planar-structure HC(NH_2_)_2_PbI_3_-based perovskite solar cells. Nat. Energy.

[57] Shi J (2015). Control of charge transport in the perovskite CH_3_NH_3_PbI_3_ thin film. ChemPhysChem.

[58] Anaraki EH (2016). Highly efficient and stable planar perovskite solar cells by solution-processed tin oxide. Energy Environ. Sci..

[59] Tan H (2017). Efficient and stable solution-processed planar perovskite solar cells *via* contact passivation. Science.

