# Supplementary material for: Manipulation of facet orientation in hybrid perovskite polycrystalline films by cation cascade
Source: Nat Commun. 2018 Jul 18;9:2793. doi: 10.1038/s41467-018-05076-w (PMC6052040; doi:10.1038/s41467-018-05076-w)
Supplement: Supplementary file 1 — Supplementary Information [file 41467_2018_5076_MOESM1_ESM.docx]

**Supporting Information**

**Manipulation of facet orientation in hybrid perovskite polycrystalline films by cation cascade**

**Supplementary Figures**


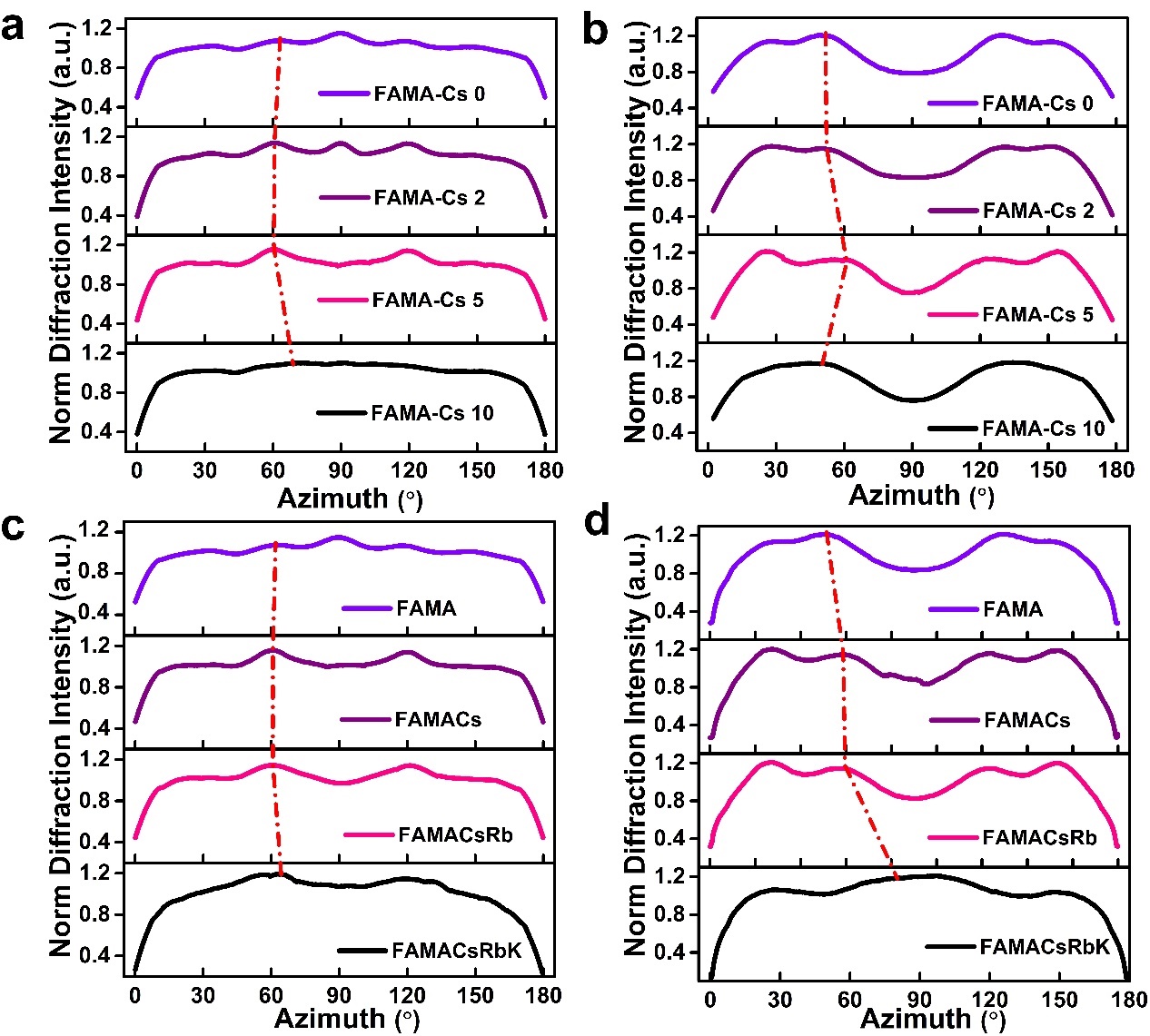


**Supplementary Figure 1 |** Integrated intensity plots azimuthally along the ring assigned to the (011) (a) and (002) (b) plane of corresponding perovskite films with Cs^+^ doping described as the label. (011) (c) and (002) (d) plane of corresponding the cation cascade doped perovskite films described as the label. See **Supplementary Note 2.**

**
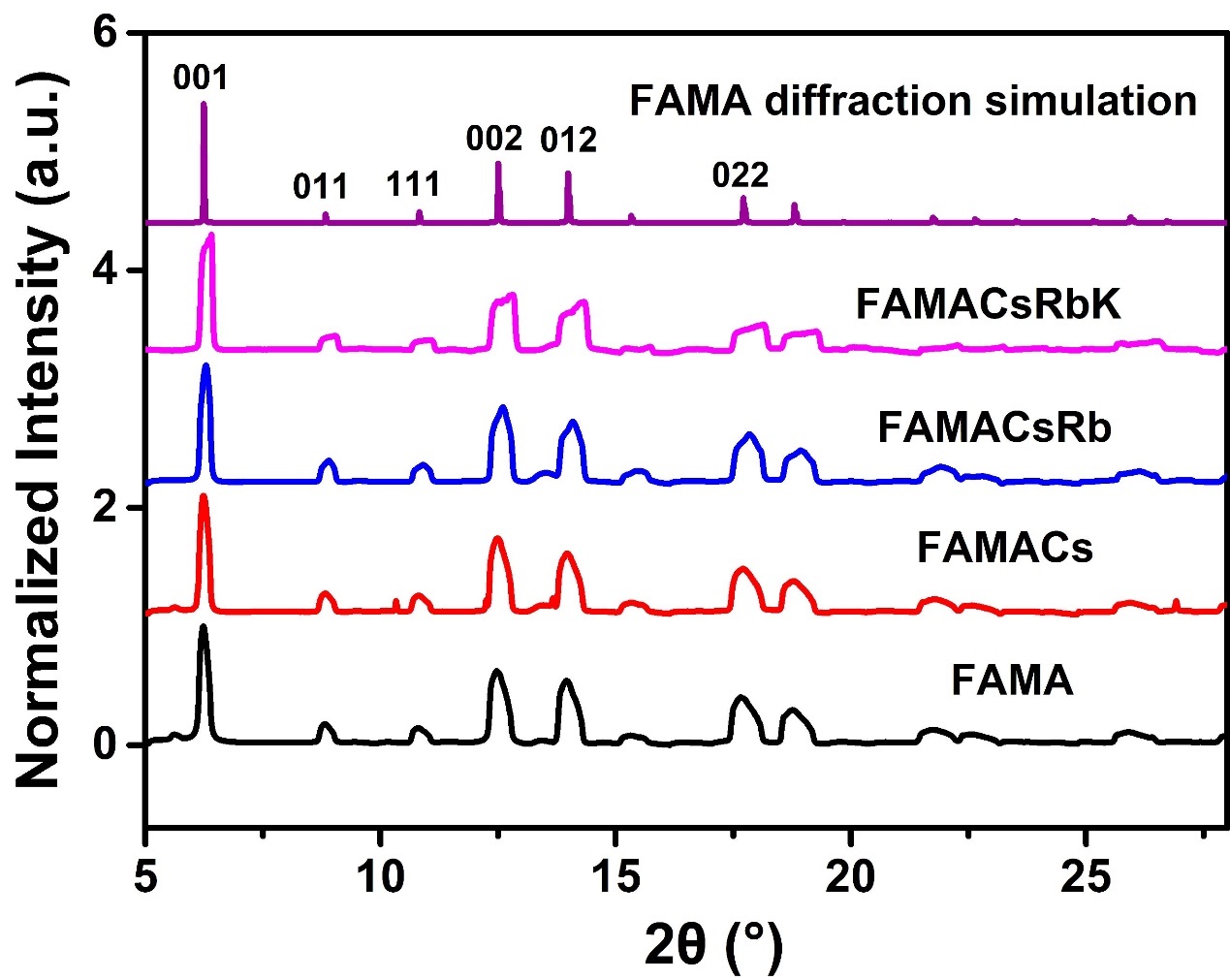
**

**Supplementary Figure 2** **|** Normalized XRD patterns of FA_0.85_MA_0.15_ diffraction simulation and the cascade doping perovskite film.


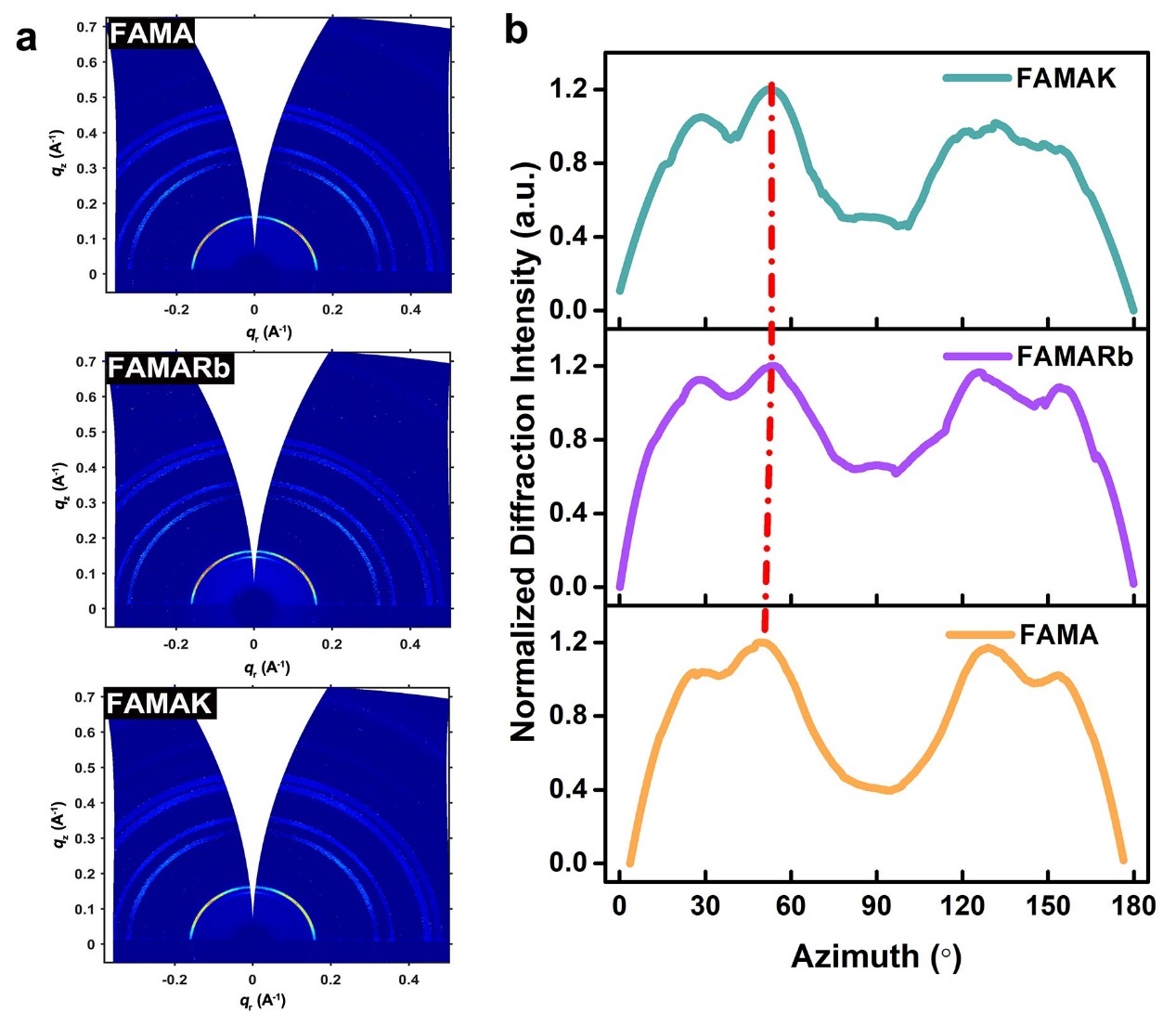


**Supplementary Figure 3| GIWAXS analysis for solely-doped samples based on FAMA.** The characterization of FAMA, FAMARb, FAMAK: a) GIWAXS patterns and b) Integrated intensity plots azimuthally along the ring at q = 10 nm^−1^, assigned to the (001) plane of corresponding perovskite films described as the label.

**
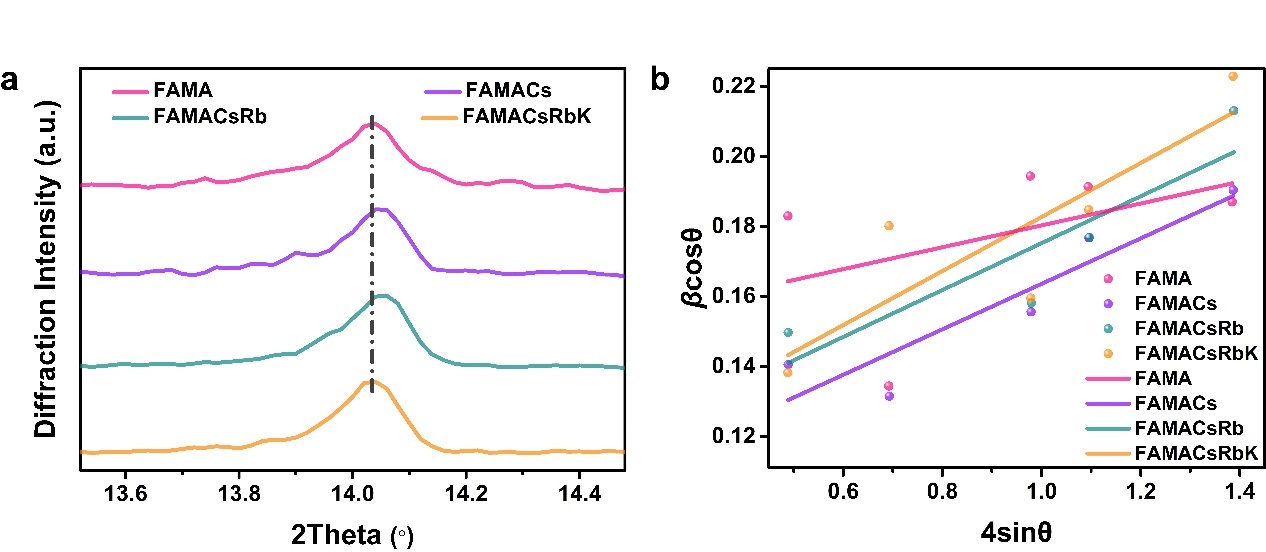
**

**Supplementary Figure 4** **| XRD and microstrain analysis.** XRD and microstrain of the perovskite films with cation cascade. See **Supplementary Note 4**.


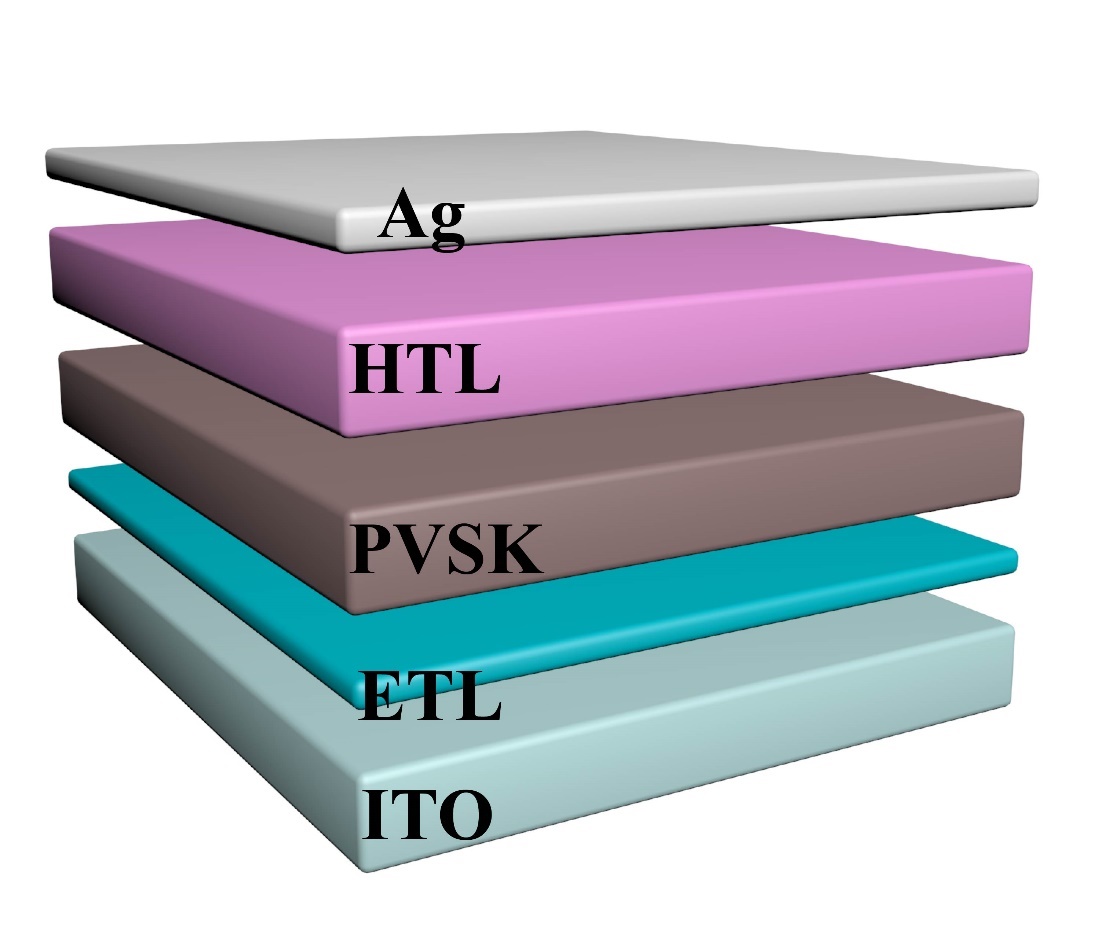


**Supplementary Figure 5** **| The configuration of solar cells.** Schematic representation of the adopted planar structure (from bottom to top): ITO/SnO_2_/Perovskite/Spiro-OMeTAD/Ag.


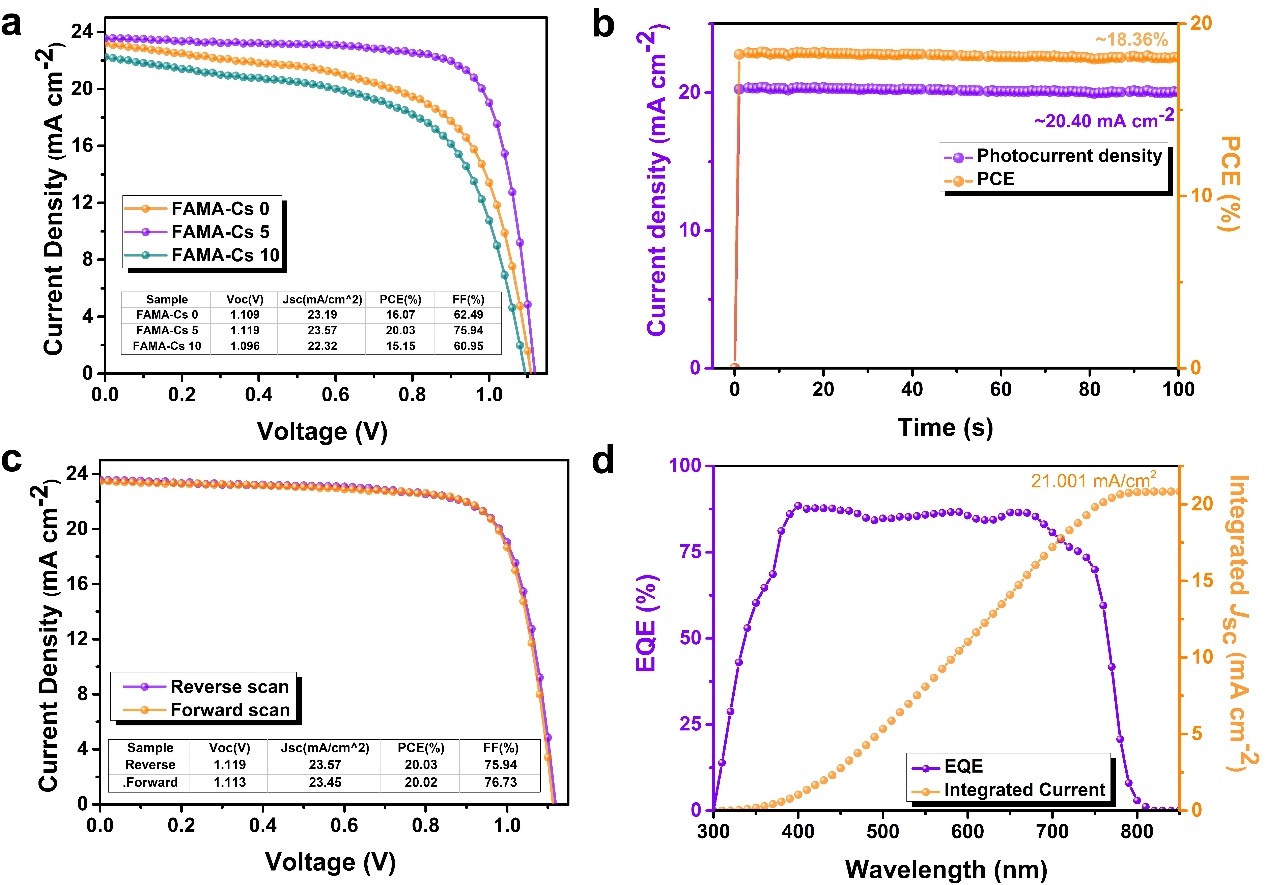


**Supplementary Figure 6** **| Device performance of perovskite solar cells.** a) Typical photocurrent density-voltage characteristics of the perovskite devices with different cesium content, including 0, 5%, 10%; b) the stable state output current density (violet) and PCE (yellow) with optimized cesium doping concentration; c) J-V curve for the champion devices under reverse (violet) and forward scan (yellow) direction; d) External quantum efficiency (EQE) of the optimized device (violet) and integrated short-circuit current density (yellow).


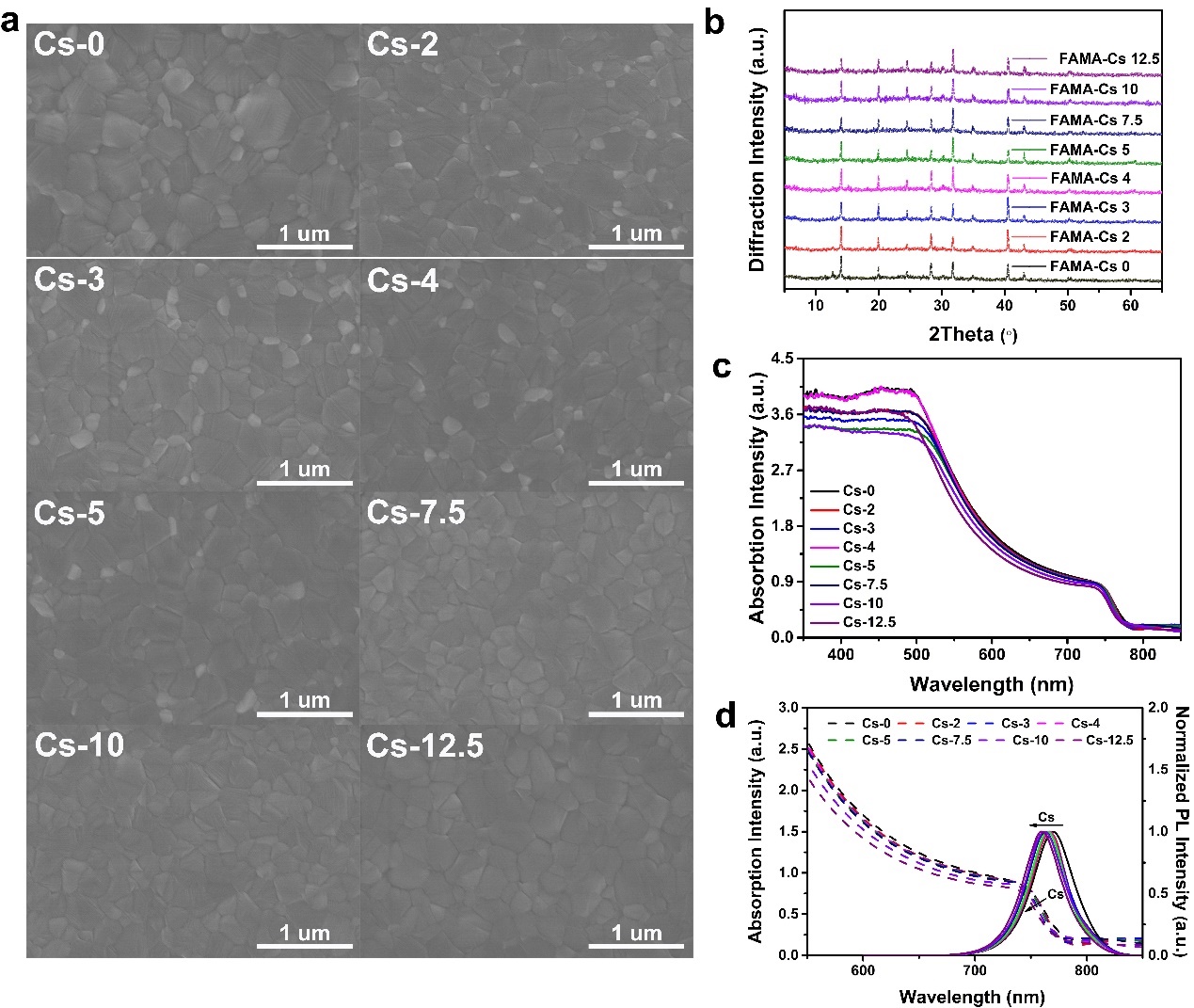


**Supplementary Figure 7** **|** Morphology, crystallinity and optical properties for perovskite films. Characterizations for morphology, crystallinity and optical properties for perovskite films depicted as (FAMA)_(100-_*_x_*_)_Cs*_x_*. The *x*=0, 2, 3, 4, 5, 7.5, 10 and 12.5 % for volume ratio. a) SEM images, b) one-dimensional XRD data and c) UV-Vis absorption spectrum for perovskite films with different cesium doping concentration depicted as the Numbers at the end of the corresponding labels; d) The corresponding absorption spectrum (dashed lines) and photoluminescence (PL) spectra (solid lines).


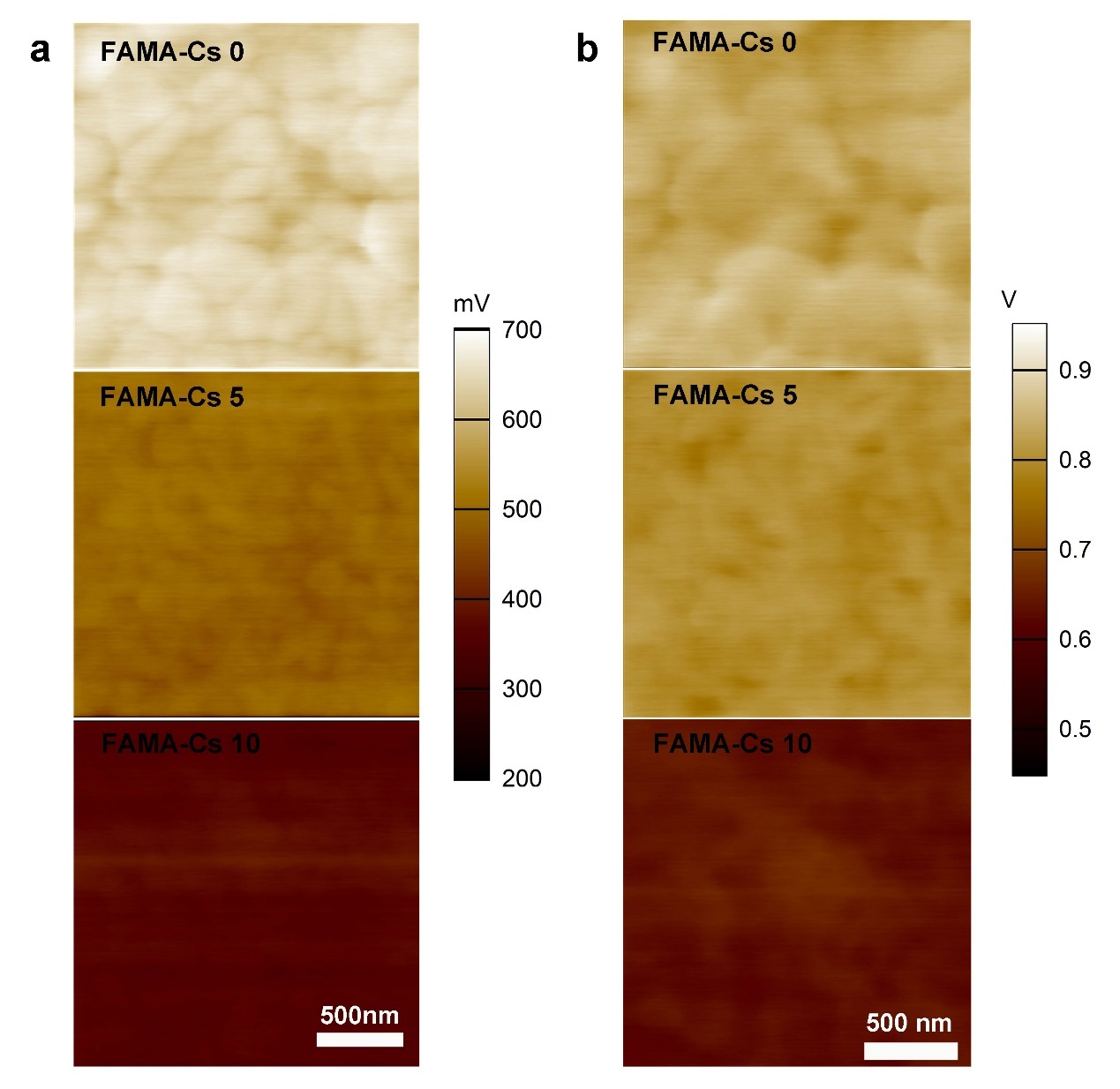


**Supplementary Figure 8** **| KPFM analyses for cesium-doped samples.** KPFM images conducted under dark (a)/light (b) conditions for FAMA perovskite films with different cesium content. See **Supplementary Note 6**.


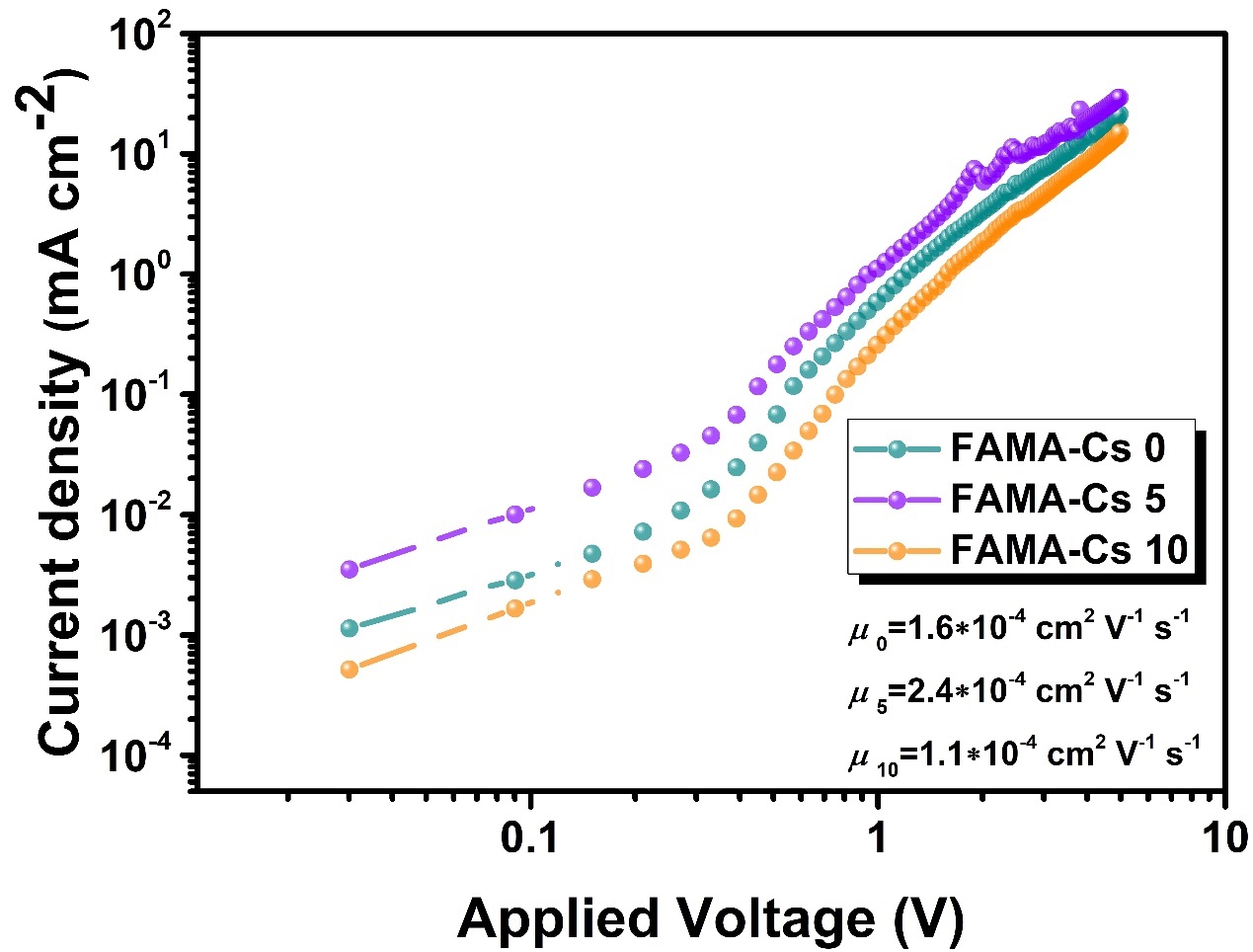


**Supplementary** Figure 9 **|** *J-V* curves of hole-dominated devices based on different perovskite films with the configuration of ITO/PEDOT:PSS/Perovskite/P3HT/Au.

**
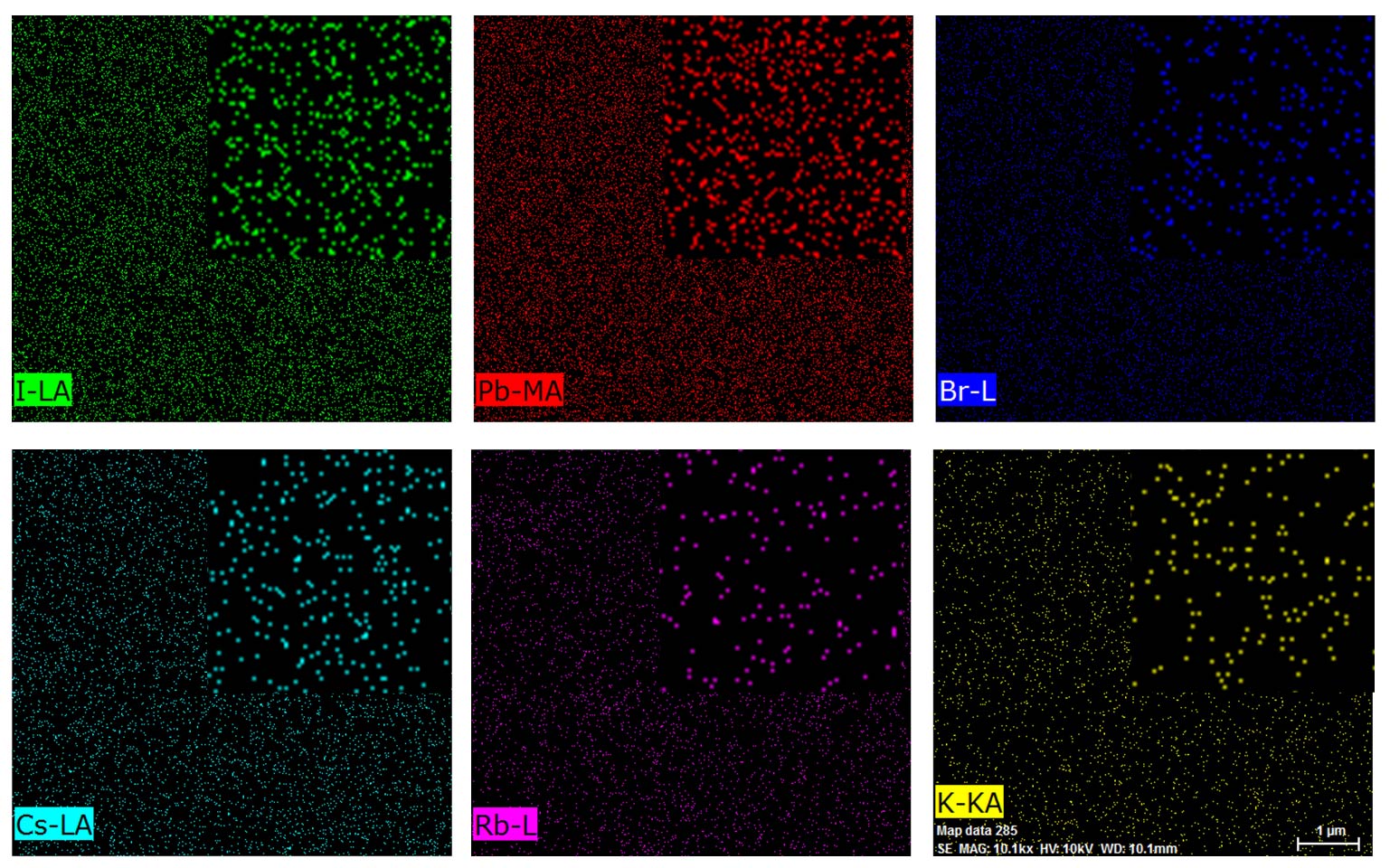
**

**Supplementary** Figure 10 **| EDX data.** EDX data for sample FAMACsRbK to explore the elements distribution. See **Supplementary Note 7.**

**
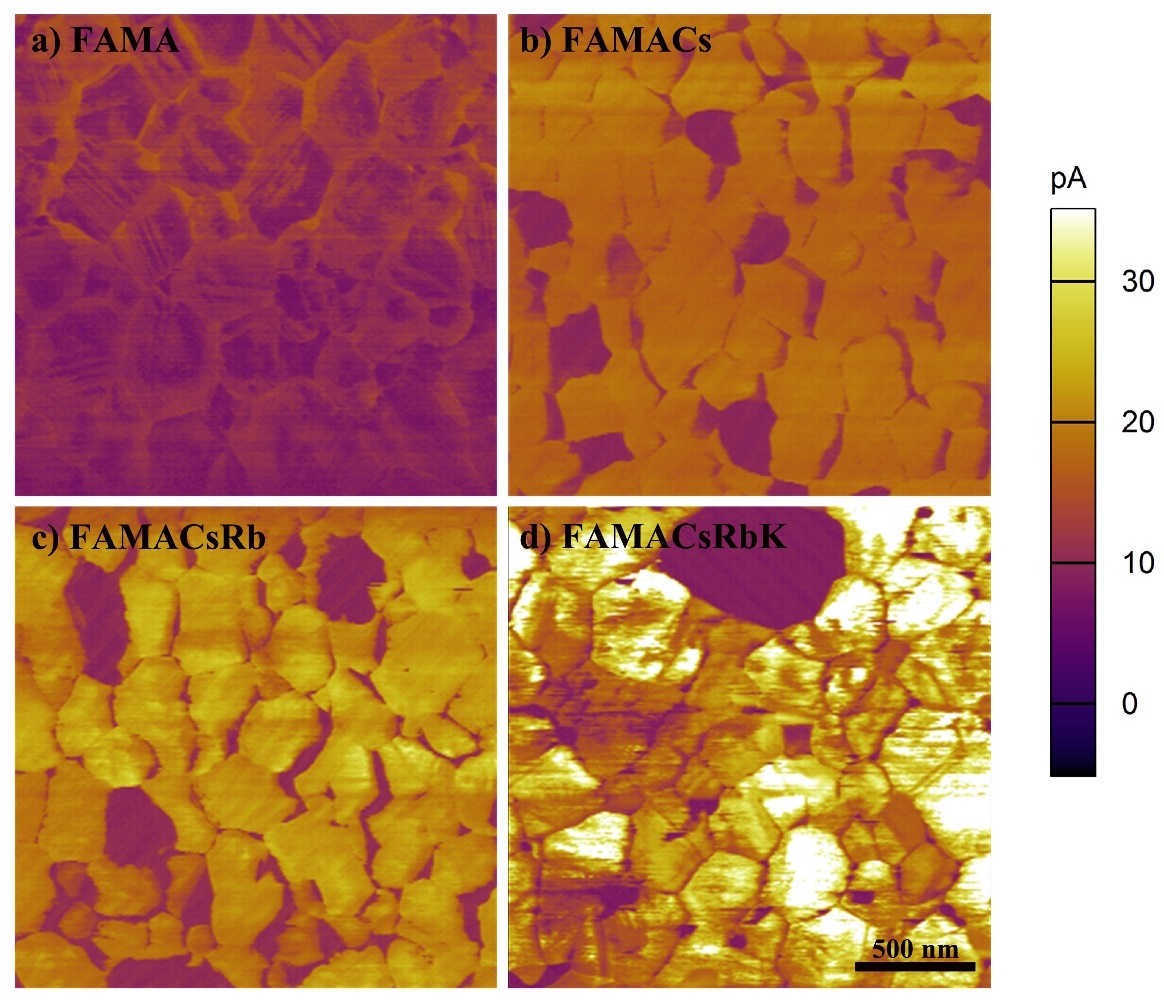
**

**Supplementary** Figure 11 **| c-AFM analysis.** c-AFM analysis for perovskites film with cation cascade doping. a) FAMA; b) FAMACs; c) FAMACsRb; d) FAMACsRbK

**
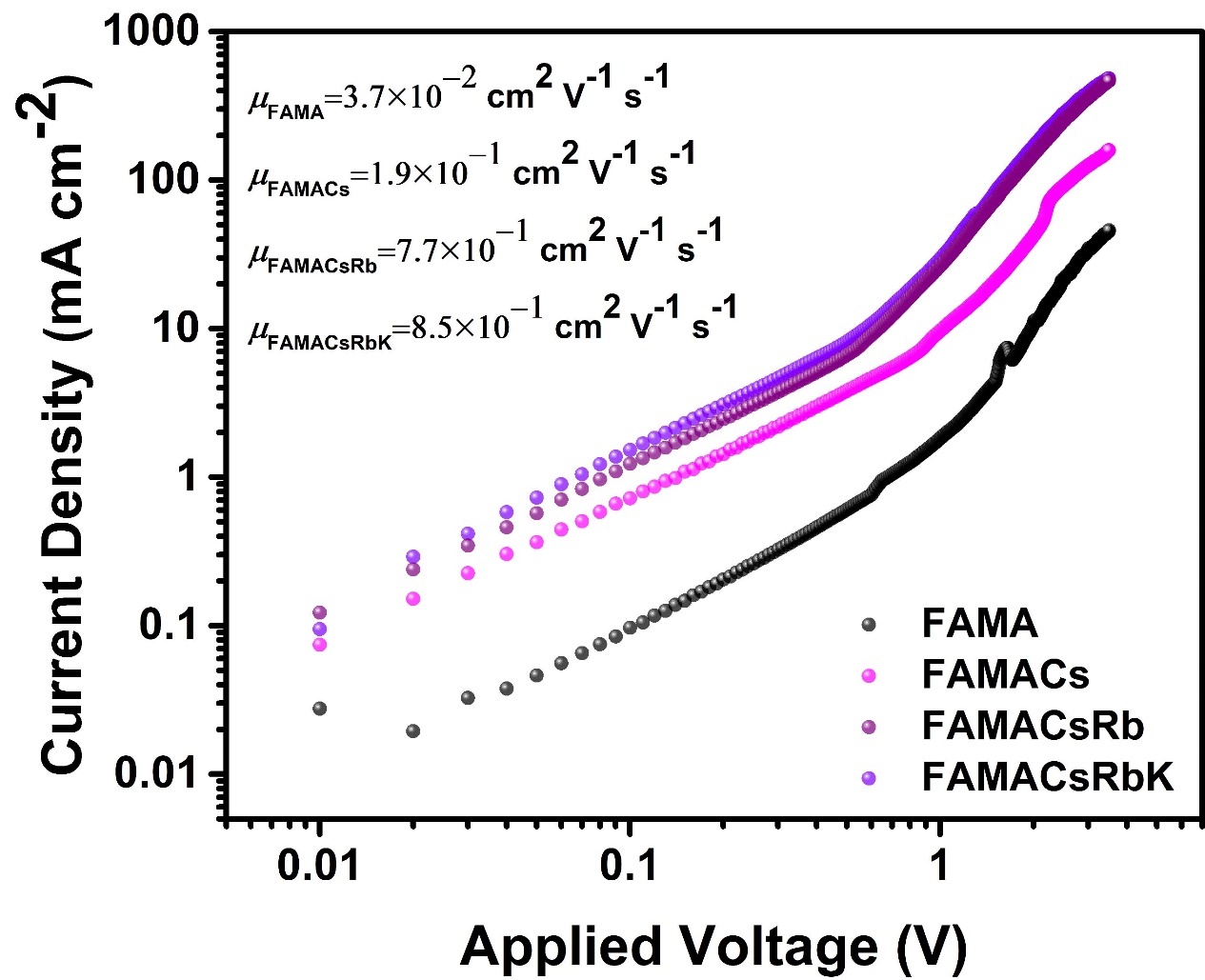
**

**Supplementary** Figure 12 **| SCLC analysis.** SCLC analysis for perovskite film with cation cascade doping. a) FAMA; b) FAMACs; c) FAMACsRb; d) FAMACsRbK.

**
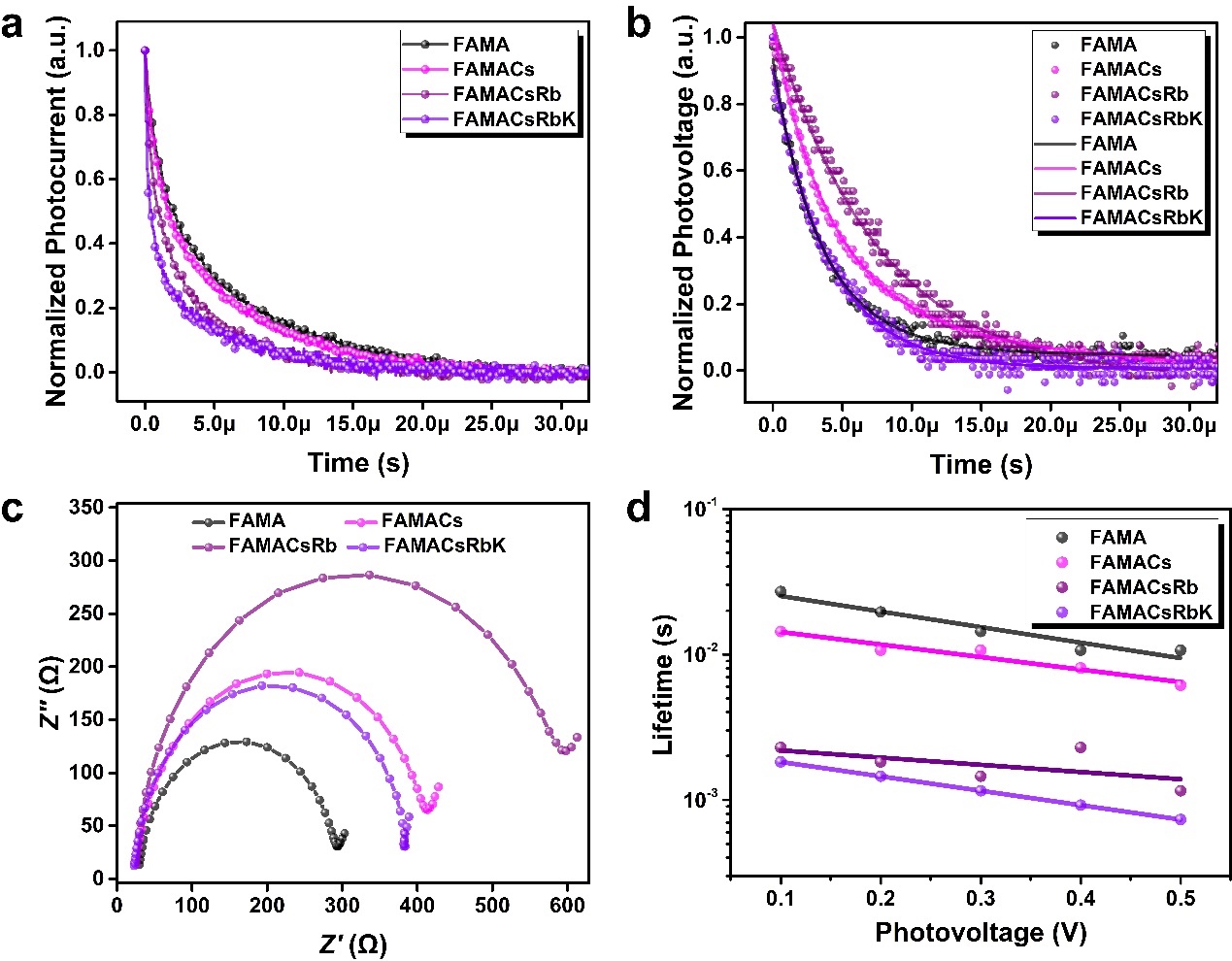
**

**Supplementary** Figure 13 **| Analysis of carrier dynamics.** a) TPC, b) TPV, c) EIS analysis and d) IMPS for FAMA perovskite films with Cs, Rb, K doping, respectively. See **Supplementary Note 9**.

**
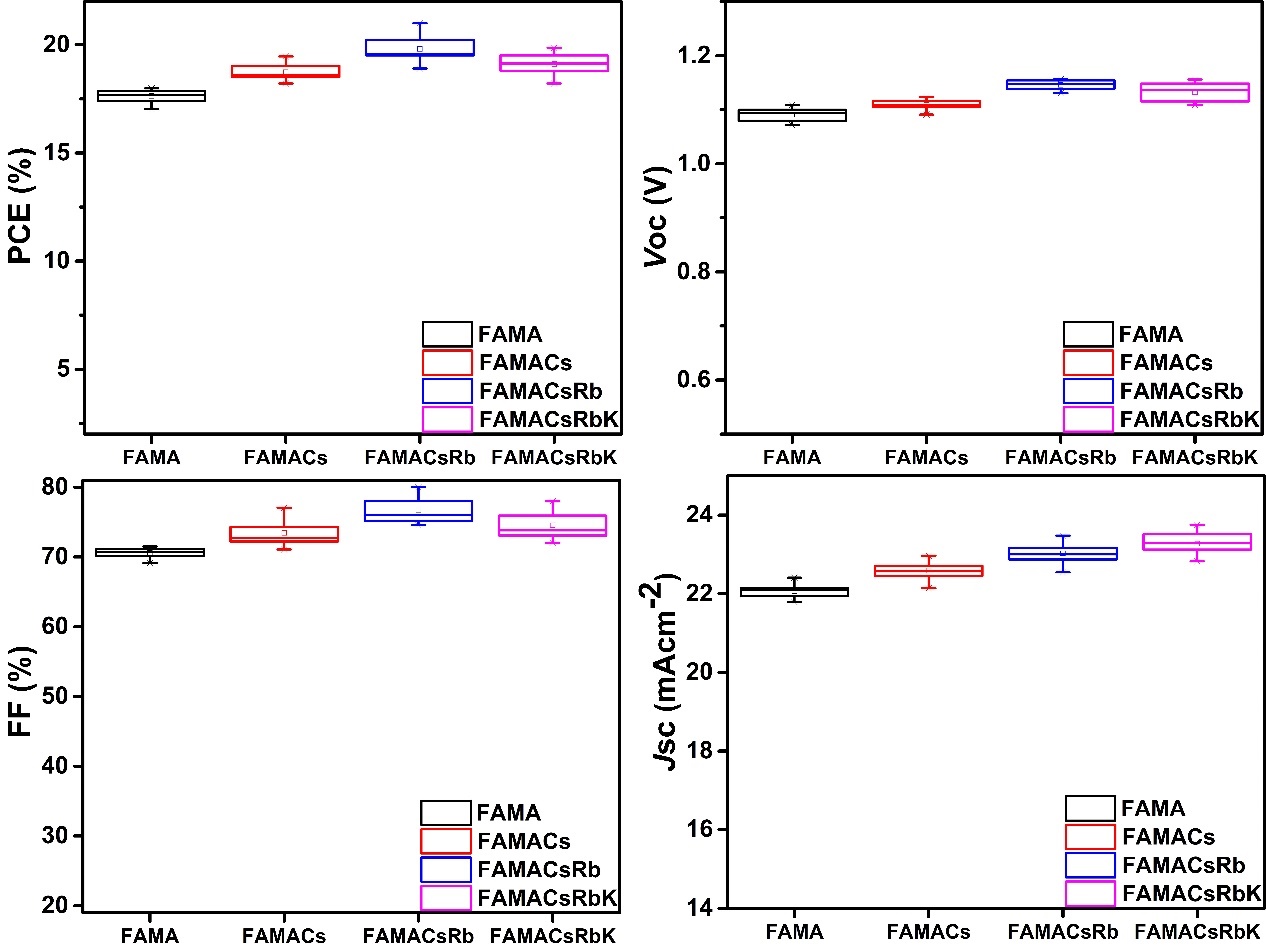
**

**Supplementary** Figure 14 **| Statistics of photovoltaic parameters.** Statistics of I-V performance parameters (*V*_oc_, *J*_sc_, PCE, FF) for devices with the scan rate of 40 mV/s based on mixed FAMA perovskites with cation cascade doping.


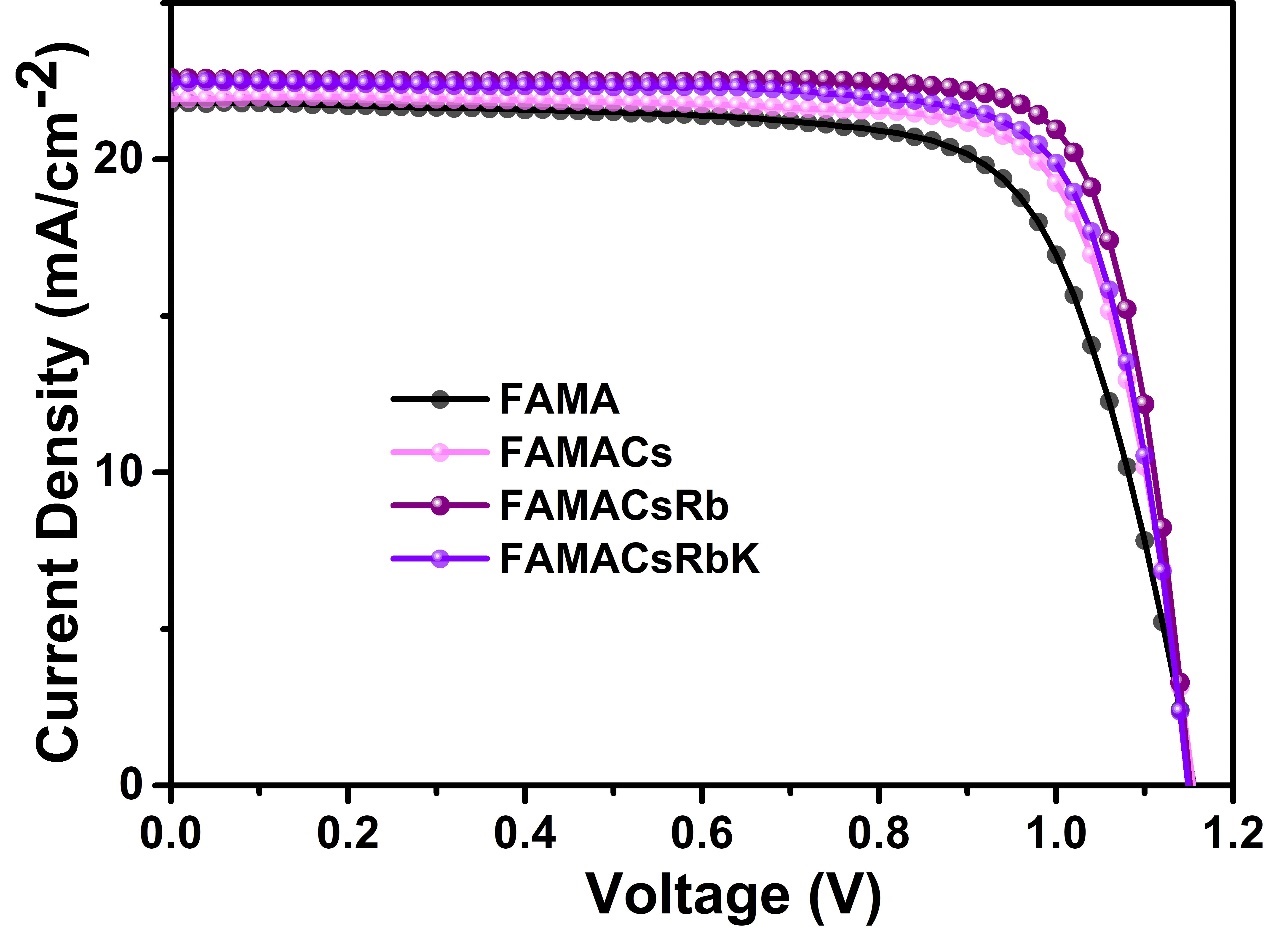


**Supplementary** Figure 15 **|** Photocurrent density-voltage characteristics of the cation cascade doped perovskite photovoltaic devices with the corresponding best performance with the scan rate of 40 mV/s.


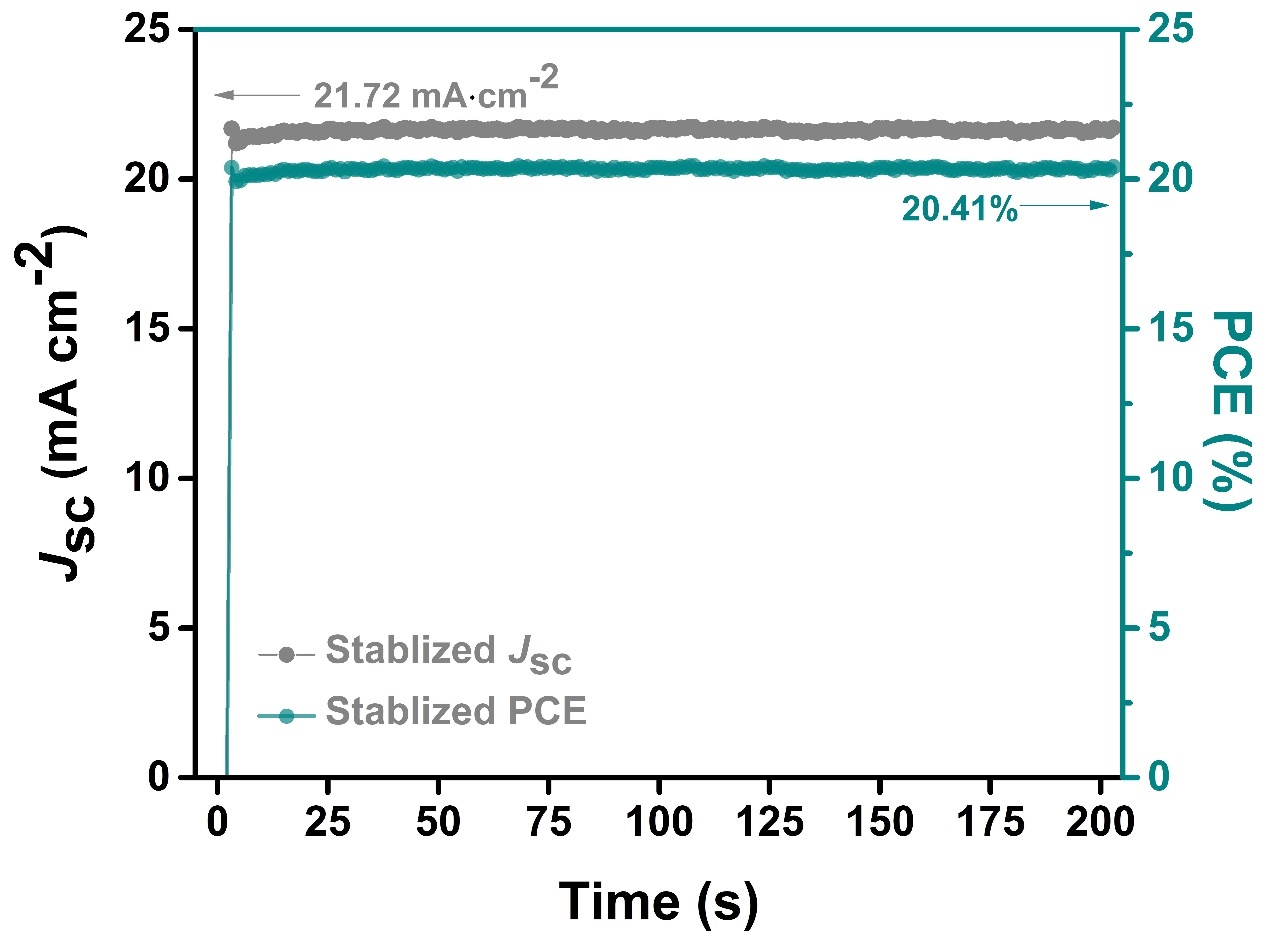


**Supplementary** Figure 16 **| Device performance of perovskite solar cells.** The steady state output current density (Grey) and PCE (Green) with cesium and rubidium cation doping. See **Supplementary Note 10**.

**Supplementary Tables**

Supplementary Table 1：The peak areas integration (**Part a**) corresponding to the different diffraction peaks in Figure 1a, and the area ratio of different diffraction peaks compared to the corresponding dominant peak (001) consisting of the same composition (**Part b**). See **Supplementary Note 1.**

| **Crystal Plane** | **(001)** | **(011)** | **(111)** | **(002)** | **(012)** | **(022)** |
| --- | --- | --- | --- | --- | --- | --- |
| **Peak position** | **14.04** | **19.94** | **24.52** | **28.3** | **31.74** | **40.51** |
| **FAMA** | **72.25** | **36.88** | **28.3** | **61.24** | **53.98** | **53.43** |
|  |  | **(011)/(001)** | **(111)/(001)** | **(002)/(001)** | **(012)/(001)** | **(022)/(001)** |
|  |  | **0.51** | **0.39** | **0.84** | **0.75** | **0.74** |
| **FAMACs** | **58.07** | **32.55** | **39.11** | **47.08** | **53.39** | **33.85** |
|  |  | **(011)/(001)** | **(111)/(001)** | **(002)/(001)** | **(012)/(001)** | **(022)/(001)** |
|  |  | **0.56** | **0.67** | **0.81** | **0.92** | **0.58** |
| **FAMACsRb** | **103.78** | **29.63** | **47.3** | **86.59** | **56.73** | **27.88** |
|  |  | **(011)/(001)** | **(111)/(001)** | **(002)/(001)** | **(012)/(001)** | **(022)/(001)** |
|  |  | **0.29** | **0.46** | **0.83** | **0.55** | **0.27** |
| **FAMACsRbK** | **93.95** | **22.59** | **47.82** | **78.64** | **58.39** | **27.15** |
|  |  | **(011)/(001)** | **(111)/(001)** | **(002)/(001)** | **(012)/(001)** | **(022)/(001)** |
|  |  | **0.24** | **0.51** | **0.84** | **0.62** | **0.29** |

**Supplementary** Table 2: The peak position corresponding to the different diffraction peaks in figure S2, and the area ratio of different diffraction peaks compared to the corresponding dominant peak (001) consisting of the same composition. See **Supplementary Note 3**

| **Crystal Plane** | **(001)** | **(011)** | **(111)** | **(002)** | **(012)** | **(022)** |
| --- | --- | --- | --- | --- | --- | --- |
| **Peak position** | **6.24** | **8.83** | **10.82** | **12.50** | **13.98** | **17.72** |
| **FAMA** |  | **(011)/(001)** | **(111)/(001)** | **(002)/(001)** | **(012)/(001)** | **(022)/(001)** |
|  |  | **0.25** | **0.28** | **1.02** | **0.98** | **0.94** |
| **FAMACs** |  | **(011)/(001)** | **(111)/(001)** | **(002)/(001)** | **(012)/(001)** | **(022)/(001)** |
|  |  | **0.23** | **0.25** | **1.09** | **0.94** | **0.90** |
| **FAMACsRb** |  | **(011)/(001)** | **(111)/(001)** | **(002)/(001)** | **(012)/(001)** | **(022)/(001)** |
|  |  | **0.22** | **0.25** | **1.05** | **0.92** | **0.92** |
| **FAMACsRbK** |  | **(011)/(001)** | **(111)/(001)** | **(002)/(001)** | **(012)/(001)** | **(022)/(001)** |
|  |  | **0.20** | **0.20** | **1.03** | **1.02** | **0.92** |
| **FAMA diffraction simulation** |  | **(011)/(001)** | **(111)/(001)** | **(002)/(001)** | **(012)/(001)** | **(022)/(001)** |
|  |  | **0.08** | **0.11** | **0.65** | **0.59** | **0.33** |

**Supplementary Table 3**: The Diffraction Peaks locations of corresponding Crystallographic planes in the perovskite films with cation cascade. See **Supplementary Note 4**.

| **Sample** | **（001）** | **（011）** | **（002）** | **（012）** | **（022）** |
| --- | --- | --- | --- | --- | --- |
| **FAMA** | 14.0225 | 19.91853 | 28.29177 | 31.74234 | 40.50693 |
| **FAMACs** | 14.03697 | 19.95315 | 28.3321 | 31.78842 | 40.5668 |
| **FAMACsRb** | 14.03838 | 19.49025 | 28.34852 | 31.80686 | 40.59968 |
| **FAMACsRbK** | 14.02547 | 19.93274 | 28.31318 | 31.77445 | 40.55815 |

**Supplementary** Table 4：Summary of devices performance parameters for typical perovskite–based devices with 0, 5% and 10% cesium content. See Supplementary Note 5.

| Sample | *V*_oc_ (V) | *J*_sc_ (mA/cm^2^) | PCE (%) | FF (%) |
| --- | --- | --- | --- | --- |
| FAMACs-0 | 1.109 | 23.19 | 16.07 | 62.49 |
| FAMACs-5 | 1.119 | 23.57 | 20.03 | 75.94 |
| FAMACs-10 | 1.096 | 22.32 | 15.15 | 60.95 |

**Supplementary** Table 5：Statistics of contact potential differences derived from KPFM images with or without illumination for perovskite films doped with 0, 5% and 10% cesium. See Supplementary Note 6.

|  | FAMA-Cs 0 | FAMA-Cs 5 | FAMA-Cs 10 |
| --- | --- | --- | --- |
| CPD_dark_ (meV) | 640 | 492 | 365 |
| Work Function_dark_ (eV) | 4.56 | 4.71 | 4.83 |
| CPD_light_ (meV) | 834 | 798 | 637 |
| Work Function_light_ (eV) | 4.37 | 4.40 | 4.56 |
| Δ CPD (CPD_light_ - CPD_dark_ , meV) | 194 | 306 | 272 |

**Supplementary** Table 6：Statistics of average photo current densities derived from c-AFM for FAMA-based samples with 0, 5% and 10% cesium content.

|  | FAMA-Cs 0 | FAMA-Cs 5 | FAMA-Cs 10 |
| --- | --- | --- | --- |
| Photocurrent density (pA) | 35 | 46 | 22 |

**Supplementary** Table 7：Fitted decay times from TPV/TPC spectra for FAMA perovskite films doped with 0, 5% and 10% cesium.

|  | FAMA-Cs 0 | FAMA-Cs 5 | FAMA-Cs 10 |
| --- | --- | --- | --- |
| TPC (*μ*s) | 1.33 | 0.35 | 2.81 |
| TPV (*μ*s) | 3.61 | 5.36 | 1.95 |

**Supplementary Table 8:** XPS data analysis for FAM, Cs, Rb and K based samples with different detecting depth on the perovskite film, 5 nm, 7.5 nm, and 10 nm. See **Supplementary Note 8**.

| **Detection depth** | | **5 nm** | **7.5 nm** | **10 nm** |
| --- | --- | --- | --- | --- |
| **FAMA** | Pb | 138.5219018 | 172.8976617 | 293.1654716 |
|  | I | 331.8416451 | 430.0379203 | 778.1769271 |
|  | I/Pb | 2.395589728 | 2.487239654 | 2.654394881 |
| **FAMACs** | Pb | 123.764926 | 185.1238504 | 189.341855 |
|  | I | 306.1494051 | 445.0605794 | 542.5644077 |
|  | I/Pb | 2.473636231 | 2.404123393 | 2.865528109 |
|  | Cs | 3.028362843 | 6.142272133 | 7.997392063 |
| **FAMACsRb** | Pb | 99.58565861 | 116.9745908 | 202.2520655 |
|  | I | 257.4677186 | 333.4591309 | 610.4733575 |
|  | I/Pb | 2.585389525 | 2.850697135 | 3.018378853 |
|  | Cs | 7.696969036 | 10.7056171 | 23.44260794 |
|  | Rb | 5.292501901 | 6.834395437 | 14.7616692 |
| **FAMACsRbK** | Pb | 128.819174 | 182.550663 | 259.320109 |
|  | I | 359.323487 | 484.398396 | 803.030729 |
|  | I/Pb | 2.789363383 | 2.65350116 | 3.096677431 |
|  | Cs | 6.00348 | 10.4850022 | 15.4228522 |
|  | Rb | 4.39643726 | 8.78554753 | 10.3021977 |
|  | K |  |  |  |

**Supplementary** Table 9：Statistics of average photocurrent derived from c-AFM for FAMA-based samples with 0, 5% and 10% cesium content.

|  | FAMA | FAMACs | FAMACsRb | FAMACsRbK |
| --- | --- | --- | --- | --- |
| Photocurrent (pA) | 10.6 | 16.4 | 18.9 | 21.8 |

**Supplementary Table 10**: Fitted decay times from TPV/TPC spectra for FAMA perovskite films with cascade doping. See **Supplementary Note 9**.

|  | **FAMA** | **Cs** | **Rb** | **K** |
| --- | --- | --- | --- | --- |
| **TPC (*μ*s)** | 1.21 | 0.89 | 0.66 | 0.45 |
| **TPV (*μ*s)** | 3.61 | 5.36 | 6.84 | 4.00 |

**Supplementary Table 11:** Summary of devices performance parameters for the cation cascade doped perovskite solar cells with champion performance, where the I-V scan rate is 40 mV/s. See **Supplementary Note 10.**

| Sample | *V*_oc_ (V) | *J*_sc_ (mA/cm^2^) | PCE (%) | FF (%) |
| --- | --- | --- | --- | --- |
| FAMA | 1.155 | 21.78 | 18.24 | 72.44 |
| FAMACs | 1.154 | 21.93 | 19.62 | 77.45 |
| FAMACsRb | 1.151 | 22.61 | 20.99 | 80.65 |
| FAMACsRbK | 1.149 | 22.47 | 20.10 | 77.84 |

**Supplementary Table 12:** Calculation results for the corresponding perovskite system using the VASP code**.** See **Supplementary Note 11**.

|  | ***E_g_* (eV)** | **Γ-X** | |  | **Γ-Y** | |  | **Γ-Z** | |
| --- | --- | --- | --- | --- | --- | --- | --- | --- | --- |
|  |  | ***m_e_^*^/m_0_*** | ***m_h_^*^/m_0_*** |  | ***m_e_^*^/m_0_*** | ***m_h_^*^/m_0_*** |  | ***m_e_^*^/m_0_*** | ***m_h_^*^/m_0_*** |
| **FAPbI_3_** | 1.46 | 1.85 | 0.32 |  | 1.51 | 0.27 |  | 0.19 | 0.73 |
| **FA_0.875_MA_0.125_PbI_3_** | 1.54 | 2.02 | 0.54 |  | 1.74 | 0.28 |  | 0.22 | 0.69 |

**Supplementary Notes**

**Supplementary Note 1.** *XRD results discussion***.**

To be clearer, we drew different color to present different information, the light grey indicates the crystal planes, the dark grey means the diffraction peak position corresponding to the crystal planes above. The blue (light and dark), pink (light and dark), yellow (light and dark), violet (light and dark) sections represent the peak area integrations (**light color, Part a**) and area ratios (**dark color, Part b**) of FAMA, FAMACs, FAMACsRb, FAMACsRbK, respectively. In order to evaluate the change in relative intensity between different diffraction peaks of the samples with cation cascade doping, we calculated the area ratio of peaks at 2θ =19.94, 24.52, 28.30, 31.74 and 40.51°, as relative to the corresponding peaks at 2θ =14.04° using Gaussian fitting as shown in Supplementary Table 1. As can be seen from the XRD pattern in Figure 1a and calculated results above, (001) and (002) are the dominant peaks for all the samples, and the area ratio of (002)/(001) was almost consistent regardless the cation cascade doping. But the relative intensity of other peaks don’t present a systematic change, for example, the peak area ratio of (111)/(001) raised from 0.39 to 0.67 with the Cs^+^ doping, but dropped to 0.46 with CsRb co-doping, and finally raised back to 0.51 for the FAMACsRbK sample. Considering the susceptibility of perovskite materials to the fabrication condition, the fluctuations of area ratios for different peaks may be caused by the accumulative fluctuation during film fabrication. Nevertheless, the cascade doping didn’t change the dominant phase and crystal orientation (001) for samples with different compositions.

**Supplementary Note 2.** *Analysis of microstructure variations for different samples.*

As can be seen in **Supplementary** **Figure 1a**, the (011) plane within FAMA films showed an obvious peak at the azimuth angle of 90^ο^, and two looming convex at 63^ο^ and (180-63) ^ο^. With the introduction of optimized concentration of cesium, these diffraction mottling peaks became more obvious and moved towards low azimuth angle from 63° to 60°. But when the concentration of Cs^+^ was increased to 10%, the diffraction mottling peak instead moved back to an even higher azimuth angle about 73°. The plane stacking pattern for (011) showed a similar tendency but an opposite direction to (001) plane. This may originate from the correlation between (001) and (011) planes, where the shifting of (001) plane will affect the arrangement of another set of crystallographic planes. In addition to (011) plane, we have also analyzed the diffraction molting peaks of (002) plane (**Supplementary** **Figure 1b**). As expected, the orientation of (002) plane shows similar trend as that of (001) planes (**Figure 3a**), which may relate to the same family of crystal planes. The similar trend has been also observed in the perovskite film with cation cascade doping, as shown in **Supplementary** **Figure 1c** and **1d.** From the above analysis, we suggested that certain texture existed in the inner grain, and different orders of diffraction are correlated. It is believed that the oriented attachment along a specific crystallographic orientation may trigger the sequential organization of neighbouring crystal plane, to reduce the overall energy.

**Supplementary Note 3.** *Integration of Debye rings from GIWAXS data.*

We integrated the intensity of Debye rings from GIWAXS data to obtain the diffraction intensity versus 2-theta. The fixed intensity ratios expect from theory is shown in the XRD pattern by powder diffraction simulation. (see **Supplementary Figure 2**) We can figure out the cation cascade doped perovskite film shows preferred orientation of (001) plane as compared to the simulation results. Subsequently, the relative intensities of high orders with respect to the first order (001) were calculated as summarized in **Supplementary Table 2**. To be clearer, we drew different color to present the corresponding information, where the light grey indicates the crystal planes, the dark grey means the diffraction peak position corresponds to the crystal planes above. The blue-, pink-, yellow-, orange- color coded sections represent the peak area ratios (the higher order planes versus (001) plane) of FAMA, FAMACs, FAMACsRb, and FAMACsRbK, respectively. We observed that the relative intensity decreased, but slightly, in most higher orders planes upon cation cascade doping. Moreover, the deviation of intensity ratio (high order plane versus (001) plane) in the perovskite films with cation cascade doping didn’t change much. Given the similar integral area of (001) peak in each sample, it indicates the series of perovskite films bear the comparable crystal quality and preferable growth plane.

**Supplementary Note 4.** *XRD and microstrain analysis.*

As can be seen in **Supplementary Figure 4a and Supplementary Table 3**, a set of diffraction peaks located at 14.02^ο^, 19.92^ο^, 28.19^ο^, 31.74^ο^, 40.51^ο^ (corresponding to different crystallographic planes) slightly shift towards large angle. It clearly indicates the decrease of lattice parameter within the cascade doped samples, from FAMA, FAMACs, to FAMACsRb. It is also observed that the shift of diffraction peak from FAMACs to FAMACsRb sample is smaller when compared to that from FAMA to FAMACs sample. This suggests that 5% Cs can be readily integrated in the crystal lattice of FAMA sample, but the tendency for Rb doping is smaller. Nevertheless, the subtle shift of diffraction peak from FAMACs to FAMACsRb is still an indicator that Rb can partially come into the crystal lattice in the present circumstance (doping condition). By further analyzing the diffraction peak in FAMACsRbK sample, we found the diffraction peak is shifting back to lower angle, which suggests that K may not be able to be inserted into the crystal lattice. Moreover, the involvement of K cation affects the integration of Cs and Rb in the FAMA crystal lattice. It obeys a fundamentally different mechanism in contrast to that of Cs and Rb cations doping, which is further investigated by comparing the microstrain derived from XRD profiles.

The microstrain is another useful parameter to describe the lattice distortion within the crystal. In general, the broadening of diffraction peaks has two main causes, including crystal size and microstrain in crystals. Using Williamson-Hall methods to handle XRD data^[1]^, crystal imperfections and distortion can be revealed by strain-induced peak broadening, according to ε≈βs/tanθ. The relationship between full-width half maximum of diffraction peaks and microstrain in crystals can be expressed using the following equations:

$$\beta_{hkl}=\beta_{S}+\beta_{D}$$

$$\beta_{hkl}=\left( \frac{k\lambda}{D\cos\theta} \right)+4\varepsilon\tan\theta$$

where ε is the microstrain, *β*s and *β*_D_ is the full-width half maximum of diffraction peaks induced by microstrain and grain size respectively, k is a dimensionless shape factor, λ is the X-ray wavelength, D is the mean size of the ordered (crystalline) domains, which may be smaller or equal to the grain size. According to:

$$\beta_{hkl}=\left( \frac{k\lambda}{D} \right)+4\varepsilon\sin\theta$$

we know that the steeper of the slope, the larger the microstrain. The microstrain in the corresponding perovskite films are shown in **Supplementary Figure 4b**.

Clearly, the cation cascade doping from Cs, Rb, K enables a monotonically increasing microstrain in the resultant films. Notably, the FAMACsRbK films exhibits the largest microstrain, although the K cannot insert into the crystal lattice of the FAMACsRb perovskite film. This suggests that cation incorporation in series could result in the increased microstrain in the film, regardless of the occupying sites, *e.g*. in the crystal lattice, or the interstitial position, or at grain boundaries or interfaces. It is reasonable to infer that the series introduction of alkalis elements leads to the higher degree of lattice distortion, and the resultant microstrain within the crystals contributes to guide the orientation variation in the polycrystalline films (**Figure 2** in the manuscript). This further reinforces the argument in our manuscript, that the cation cascade doping could lead to the lattice distortion, and eventually to the orientation variation.

Supplementary Note 5. *Device performances for samples with different cesium doping conditions.*

From Supplementary Table 4, we observe that certain amount (5%) of cesium doping enhanced the device performance significantly. However, when the Cs^+^ amount was increased to 10%, the device performance decreased substantially to a value even lower than the reference. To be noted, the intensity of PbI_2_ in the doped FAMA film decreased systematically with enhanced cesium concentration, as seen in **Supplementary Figure 7b**. The absence of a monotonic relationship between efficiency and PbI_2_, suggests that PbI_2_ is not the key parameter that affect the device performance. According to other reports, that the presence of PbI_2_ residual in the perovskite film affects the photovoltaic performance of the resulting device in both ways. On the other hand, we have found that the excessive PbI_2_ can’t change the crystal stacking direction which will be further reported in our sequential work. Therefore, in a word, the obvious enhancement in PCE is not resulted from the quantitative difference in excessive PbI_2_.

**Supplementary Note 6.** *Local surface potentials analysis.*

KPFM measurements were conducted with/without illumination using a Dimension Icon Scanning Probe Microscope (Bruker Nano, Inc.) with a Pt-coated silicon probe and provided reliable information of local surface potentials stemming from contact potential differences (CPD) between the tip and sample surface associated with their relative work functions. Spatial maps of local surface potential for samples on ITO substrate with varied doping conditions were presented in **Supplementary** **Figure 8**, with the increased cesium content from 0% (reference sample), 5% (the optimized doping concentration), to 10%. The corresponding averaged CPD decreased systematically from 640 meV to 521 meV, 492 meV, indicating that Fermi energy level within perovskite thin films declined gradually. In addition, the incorporation of cesium can also slightly enlarge the band gap of perovskite thin films, as shown in the UV-vis absorption and PL spectra. It leads to the downshift of fermi level of Cs^+^ doped perovskite and modulated the corresponding band energy level alignment. To be more visualized, we also carried out the KPFM measurements for each sample under illumination and compared the obtained results with and without illumination as discussed in the above. The corresponding CPD differences of the samples were shown in **Supplementary** **Table 5**. It was found that sample with the optimized doping concentration (5%) showed the largest Δ CPD under the condition of with or without illumination among all samples. This suggests a different photoresponse in the perovskite thin films, and the optimized Cs^+^ in the FAMA film leads to an enhanced photoresponse. It could be deduced that the variation of cesium-doping could systematically modulate the photoresponse of perovskite, presumably ascribed to the interior alteration in microstructure.

**Supplementary Note 7.** *Elements distribution analysis.*

As can be seen in **Supplementary** Figure 10, all the elements of I, Pb and Br distributed homogeneously. The doping elements of K, Rb and Cs showed lower signal intensity due to their restricted concentration in the starting materials. Besides, K content that detected by EDX was less than Cs or Rb as indicated from the EDX mapping. In addition, when mapping of Cs, Rb and K, there were certain blank areas where the corresponding elements can’t be detected. By combining this phenomenon with the XRD results, we speculated that only certain amount of alkalis elements incorporated into the lattice, leaving the rest residing in the surface/grain boundaries of the films. Due to the limited resolution, we can only provide the qualitative analysis by SEM characterization.

**Supplementary Note 8.** *Angle-resolved XPS measurement with different detecting depth.*

We carried out angle-resolved XPS measurement with different detecting depth on the perovskite film, roughly 5 nm, 7.5 nm, and 10 nm respectively. The detailed analysis of the four samples was provided in **Supplementary** **Table 8**. Accordingly, it was observed that the I/Pb ratio is slightly increased to or close to 3 by increasing the detecting depth, in all the four samples. This was consistent with other study ^[2]^ to confirm that the PbI_2_ are likely to locate at the surface of the perovskite film. Mostly interestingly, it was observed that the Cs, Rb were detectable on the surface, while K content was below the detection limit. The content of Cs in the FAMACs, FAMACsRb and FAMACsRb sample, as well as the Rb in FAMACsRb and FAMACsRbK sample are gradually increased along the detection depth. We also found that the Cs content in the corresponding film is increased by over 2 times when the detection depth was increased from 5 nm to 10 nm, which suggesting that the Cs is more likely to exist in the bulk. Similar to the Cs doping, Rb doping also follows the similar trend with increased amount in the bulk. This suggests that the Cs and Rb stay in the polycrystalline films to some extent. The absent of K on the surface is not fully understood, which will be further investigated in the following work.

**Supplementary Note 9.** *Transient photocurrent or photovoltage measurements.*

TPC and TPV measurements can provide a transient photocurrent or photovoltage response excited by a short laser pulse, which are widely employed to probe the photo-carrier lifetime related to carrier kinetics in the perovskites solar cells. TPC measurements were conducted on the devices based on FAMA, FAMACs, FAMACsRb, and FAMACsRbK (**Supplementary** Figure 13a and **Supplementary Table 10**). The FAMACsRbK based device exhibited the shortest decay time of 0.45 *μ*s, standing for the fastest charge transport process as compared with that of FAMACsRb, FAMACs and FAMA based devices (0.66 *μ*s, 0.89 *μ*s, and 1.21 *μ*s, respectively). The decrease in photocurrent decay time in these cells were observed upon cation cascade doping, indicating that the cascade doping effectively promoted the charge extraction and transport process which correlated perfectly with the orientation variation. In contrast, the TPV results for these samples don’t follow the same pattern to the TPC results. As shown in **Supplementary Figure 13b**, the photovoltage decay time gradually increased first, namely, about 3.61 *μ*s (FAMA), 5.36 *μ*s (FAMACs), and 6.84 *μ*s (FAMACsRb), but it unexpectedly decreased to about 4.0 *μ*s (FAMACsRbK). As photovoltage decay time describes carrier recombination kinetics, it indicates that the underlying mechanism to govern carrier recombination may be complex. Cation cascade in this case may introduce not only crystal orientation change but other effects, and they both affect the carrier recombination process at the interface.

In addition, we probed the photo-carrier dynamics from the electrochemical perspective, wherein electrochemical impedance spectroscopy (EIS) characterization was carried out to demonstrate carrier transport processes at the interface. It is generally accepted that the middle frequency zone of EIS semicircle is considered to be dominated by junction capacitance and recombination resistance that associated to the interfaces between carrier transport layer and the absorber. As shown in **Supplementary Figure 13c**, the FAMACsRb based device has the largest impedance among the three samples, followed by FAMACs, FAMACsRbK and FAMA. This is in excellent agreement with the above TPV results, where FAMACsRb instead of FAMACsRbK based sample presents a substantial suppressed recombination.

To further explore the influence of the cation cascade, the intensity-modulated photocurrent spectroscopy (IMPS) was employed to estimate the charge transport lifetime (*τ*) of the devices under different illumination intensity. *τ* can be derived from the expression: *τ*= 1/2*πf*, whereas *f* is the characteristic frequency at the minimum of the IMPS imaginary component. ^[3]^ As shown in **Supplementary** Figure 13d, the τ is monotonically decreased from FAMACsRbK, to FAMACsRb, FAMACs, and FAMA, suggesting that the charge transport lifetime of the devices have been influenced by the crystal orientation even under different illumination intensity.

Till now, we found that the charge extraction efficiency, and the charge transport (or collection) time followed the similar trend. All these three results were correlated tightly with orientation variations. Combined the full characterization including GIWAXS, *c*-AFM, SCLC, TPC, TPV, EIS, IMPS, we argue that the incorporation of Cs, Rb, K take effects to boost the carrier transport not only in the perovskite film, but also in the interfaces between perovskites and transport materials, which probably associated to the crystal stacking toward the in-plane and out of plane.

**Supplementary Note 10.** *Device performance comparisons***.**

Since the device optimization on the Rb and K doped perovskites would provide more convincing evidence regarding the relationship between the intra-grain crystal orientation and optoelectronic properties in the cascade doping perovskites. Accordingly, devices based on mixed perovskites with cation cascade were fabricated and investigated. We fixed the molar ratio of FAPbI_3_:MAPbBr_3_ at 85:15 with optimized Cs^+^, Rb^+^, K^+^ doping concentration, and implemented the as-prepared absorber in devices with the common configuration of ITO/SnO_2_/Perovskite/Spiro/Ag (**Supplementary** Figure 5). The power conversion efficiency (PCE), open circuit voltage (*V*_oc_), short circuit current (*J*_sc_), and fill factor (FF) of the champion devices are summarized in **Supplementary Table 11**. It was found that the optoelectronic parameters, particularly *V*_oc_ and FF are further increased with Rb^+^ and K^+^ doping, while the *J*_sc_ is slightly decreased. Accordingly, the average power conversion efficiency (PCE) increased from 17.63% to 18.74% and 19.75%(With a champion PCE of 20.99% and stabilized efficiency of 20.41%, see **Supplementary Figure 16**.), for FAMA, FAMACs and FAMACsRb, respectively. For FAMACsRbK based sample, the corresponding average PCE (19.11%) is slightly lower than that of Rb, but higher than that of Cs based devices. These results clearly indicate that the incorporation of Rb and K enhances the performance of the mixed perovskite-based photovoltaic devices effectively. As indicated in the GIWAXS measurement, the (001) crystal plane has the tendency from *α* orientated stacking to both the in-plane and out-of-plane with the cation cascade doping, *e.g.* FAMA, FAMACs, FAMACsRb, FAMACsRbK, as well as the enhanced long-range orienated crystallization. Therefore, it is also consistent with the fact that the optoelectronic properties can be modulated upon A site cation cascade doping partially governed via manipulation of crystal facet orientation, which provides an alternative way to boost the device performance.

**Supplementary Note 11.** *Calculation results using the VASP code.*

We calculated the effective masses of electrons and holes of the archetype FAMAPbI_3_ materials, by using the Vienna Ab initial Simulation Package (VASP) code in the framework of density functional theory (DFT). The electron-ion interaction was described by the projector augmented wave (PAW) method. The plane-wave cut-off energy was set to 450 eV. The Monkhorst-Pack k-point meshes of 3×3×3 were chosen for sampling the Brillouin zone. The lattice parameters and atomic positions were fully relaxed until the force on each atom was smaller than 0.03 eV/Å. The starting geometry of FAPbI_3_ is taken from the experimental structure. FA_0.875_MA_0.125_PbI_3_ was modeled using the 2×2×2 supercell with 96 atoms. For structural relaxation, the generalized gradient approximation (GGA) formulated by Perdew, Burke, and Ernzerhof (PBE) was chosen as the exchange-correlation functional. The band structures were calculated using PBE without spin-orbit coupling (SOC) method. The results were presented in **Supplementary Table 12** and these results indicate that perovskite thin films were anisotropic, the effective mass varied obviously to the crystal orientation index. To be noted, the electron effective mass showed a substantially smaller value along the **Z** direction (perpendicular to the perovskite (001) plane) for both FAPbI_3_ and FA_0.875_MA_0.125_PbI_3_ sample. Therefore, the orientation variations inevitably lead to different optoelectronic properties as evidenced in the samples with cation cascade doping. In comparison, the value of hole effective mass along different direction was less changed, indicating that the orientation variation has less impact on the hole transportation property in the perovskite film. In another word, the varied electron transportation in the perovskite film is mainly responsible for the difference in optoelectronic properties.

According to GIWAXS data, it shows that the cation cascade doping manipulates the crystal orientation, wherein (001) crystal plane stacks parallel to the substrate. As the simulation indicates the electron effective mass along the **Z** direction has the lowest value, the most efficient electron transport occurs along <001> direction in the perovskite film. It is thus expected that cation cascade doping leads to the crystal plane stacking favour for electrons transport, which is in good agreement to the SCLC measurement and PCE results. Besides, the adjustment of the orientation along the out-of-plane direction may also increase the effective contact area between (001) plane and carrier transport layers, which can promote the charge extraction process.

**Supplementary References**

[1] Prabhu, Y.T., K.V. Rao, V.S.S. Kumar*, et al.*, X-Ray Analysis by Williamson-Hall and Size-Strain Plot Methods of ZnO Nanoparticles with Fuel Variation. *World J. Nano Sci. Eng.*, 2014. **04**(01):21-28.

[2] Shih, M.C., S.S. Li, C.H. Hsieh*, et al.*, Spatially Resolved Imaging on Photocarrier Generations and Band Alignments at Perovskite/PbI2 Heterointerfaces of Perovskite Solar Cells by Light-Modulated Scanning Tunneling Microscopy. *Nano Lett*. 2017. **17**(2):1154-1160.

[3] Chen, Y., L. Li, Z. Liu*, et al.*, Photon management for efficient hybrid perovskite solar cells via synergetic localized grating and enhanced fluorescence effect. *Nano Energy*, 2017. **40**:540-549.
